# Probiotics, Prebiotics, and Synbiotics in the Irritable Bowel Syndrome Treatment: A Review

**DOI:** 10.3390/biom11081154

**Published:** 2021-08-04

**Authors:** Agnieszka Chlebicz-Wójcik, Katarzyna Śliżewska

**Affiliations:** Institute of Fermentation Technology and Microbiology, Department of Biotechnology and Food Sciences, Lodz University of Technology, Wólczańska 171/173, 90-530 Łódź, Poland

**Keywords:** probiotics, prebiotics, synbiotics, irritable bowel syndrome

## Abstract

Irritable bowel syndrome is not a life-threatening disease, yet it significantly affects the quality of life and contributes to economic loss. It is estimated that even up to 45% of the world’s population can suffer from the disease. The first attempts to diagnose irritable bowel syndrome were made at the end of the 19th century; however, establishing appropriate diagnostic criteria and treatment methods is still ongoing. To date, little is known about the etiology of irritable bowel syndrome; however, growing attention is drawn to the intestinal microbiota as a factor in the disease development. For this reason, researchers have conducted many studies on therapies that modulate the microbiota, among which probiotics, prebiotics, and synbiotics are widely studied. To date, most studies have examined probiotics; however, there are also several studies demonstrating the efficacy of prebiotics and synbiotics. The aim of this review was to summarize findings on the usefulness of probiotics, prebiotics, and synbiotics in the treatment of irritable bowel syndrome.

## 1. Introduction

Irritable bowel syndrome (IBS) is an intestinal functional disorder that is classified as a non-life-threatening disease. It causes a decline in life quality and abates an ability to function in society, as well as attributes to economic losses [1,2]. The cost of IBS is estimated to be up to EUR 8 billion in Europe, nearly USD 2 billion in China, and up to USD 10 billion in the United States of America [1]. It is predicted that up to 10% of the worldwide population suffers from this disease. Some sources estimate an even higher prevalence reaching 45% [1,3]. Women are 1.5–3 times more likely to develop IBS than men, with a two times higher possibility of constipation-associated symptoms, whereas men exhibit a diarrheal form of the condition [4]. 

Based on the manifestation of the disease, IBS is divided into four subtypes, namely, forms with predominant diarrhea (IBS-D), with prevailing constipation (IBS-C subtype), or mixed defecation types (IBS-M), as well as IBS that cannot be subdivided [5,6]. Besides altered bowel habits, IBS patients may also suffer from abdominal pain or discomfort, flatulence, nausea, dyspepsia, or reflux [7]. 

The etiology of IBS is still unknown. However, many factors could be responsible for the development of the disease. Besides psychological disturbances, the altered intestinal motility, food hypersensitivity, genetics, abnormalities of the intestinal microbiota, and impairment of the bidirectional communication pathways between the gut, its microbiota, and the central nervous system, called the gut–brain axis (GBA), could trigger the disease [8,9]. Moreover, bacterial overgrowth or post-infectious (PI) changes in the gastrointestinal tract (GIT) and inflammation are acknowledged as IBS initiators [10]. Physical or sexual abuse in childhood, a short period of breastfeeding, food allergies, obesity, or surgical interventions might also lead to the evolvement of the disease [6]. IBS patients are more prone to exhibit psychological disorders, such as anxiety or depression. This makes them less receptive to IBS treatment when it is introduced alone [11]. Therefore, psychological therapy is recommended for these individuals either as an alternative to medication or to support it [12].

Antispasmodic, antidiarrheal drugs, and laxatives are the most commonly used pharmaceuticals to treat IBS, but long-term use can lead to severe side effects [13]. Additionally, acupuncture and massage, along with traditional Chinese medicine (TCM; e.g., Huoxiang Zhengqi Soft Capsule, Guchang Zhixie Pill, Shugan Decoction, and Sishen Pill), which comprise natural plant-based ingredients, are applied as remedies for IBS [13,14]. Another promising approach to alleviate IBS is targeting the gut microbiota, for which probiotics, prebiotics, and synbiotics can be used [15]. Probiotics are defined as live microorganisms that confer a health benefit on the host, while prebiotics are substrates for the favorable microorganisms inhabiting the host’s GIT [16,17,18]. Synbiotics combine probiotics and prebiotics to improve the health effects on the host compared to using these components separately. There are two types of synbiotics: synergistic, in which prebiotic serves as a substrate for administered probiotic microorganisms, or complementary, which influence the host’s indigenous microbiota [19].

This review aimed to recapitulate the literature in the field of IBS since its early diagnosis and the first treatment attempts, as well as compile the information on the alteration of GIT microbiota among IBS individuals. Above all, the following paper intended to summarize research on the use of probiotics, prebiotics, and synbiotics in the treatment of IBS and to assess the limitations of the studies cited. 

## 2. Method

The literature search was conducted until June 2021 in the PubMed Central database. Terms: “IBS”, “Irritable Bowel Syndrome”, “Irritable Bowel”, “IBS AND Microbiota”, “Irritable Bowel AND Microbiota”, “IBS AND Probiotics”, “Irritable Bowel AND Probiotics”, “IBS AND Prebiotics”, “Irritable Bowel AND Prebiotics”, “IBS AND Synbiotics”, “Irritable Bowel AND Synbiotics” were used in the field of article’s title, abstract, as well as keywords. We included studies of all types and did not restrict the search by publication date or IBS diagnosis criteria.

## 3. History of the Illness Recognition

The history of the IBS diagnosis began in 1871 when da Costa described a condition called membranous enteritis in which patients suffered from intestinal pain accompanied by excretion of mucus [20,21]. Doctor Hale-White, who studied patients with various conditions of organic origin (e.g., colon cancer, ulcerative colitis, or appendiceal abscess), mentioned the disease at the beginning of the 20th century [22]. On the other hand, when doctor Herbert P. Hawkins (1906) analyzed reasons for the misdiagnosis of appendicitis, he concluded that there are several symptoms associated with intestines functionality, which do not have an origin in any pathological changes. The scientist speculated that constipation, diarrhea, intestinal spasms, and abdominal pain can have a nervous etiology [23]. Doctor John R. Ryle investigated a similar problem, which he termed spastic colon, as he also observed a number of unnecessary abdominal surgeries that failed to provide relief to patients suffering from chronic abdominal pain. He emphasized the vital importance of a detailed diagnosis, focusing on continuous pain, not typical for acute enteritis or bowel obstruction, as well as unusual palpability of patients’ colon caused by muscle spasm [24]. In 1937, doctor Earle P. Scarlett agreed that Ryle’s term “spastic colon” was more appropriate, rather than “colitis”, which should be used only in cases of clearly demonstrated inflammation of the colon. Additionally, the “irritable colon” appellation, first introduced by doctor Sippy, was mentioned as the equally appropriate term for this spectrum of symptoms. Although, doctor Scarlett pointed out that the disturbance of functionality might not only affect the colon but the whole intestines [25]. In the following years, IBS was found to be related to the overstimulated autonomic nervous system and patients’ personality, which, together with X-ray examinations and observations of symptomatology, led to correct diagnostics [26]. Furthermore, Misiewicz, Wallet, and Eissner (1966) observed the impact of elevated levels of 5-hydroxytryptamine (5-HT, serotonin), an amine produced in the alimentary tract, on intestinal motility resulting in diarrhea. It marked the beginning of extensive studies on the role of serotonin in the etiology of IBS [27]. Moreover, the role of the gut microbiota in IBS etiology has been extensively analyzed since the 1980s [28]. It was not until the late 1990s that gastroenteritis was found to be a contributing factor to the development of IBS [29,30]. However, the differences between the fecal microbiota of healthy individuals and IBS patients were not described until the early 2000s [31,32].

In 1962, Chaudhary and Truelove, who studied IBS as a spectrum including both mucus colitis and spastic colon, divided 130 patients into those with painless diarrhea and ones with abdominal pain, among whom Ritchie and Tuckey (1969) observed similarities in colon motor activity, although bowel function ranged from normal to diarrhea or constipation [21,22]. Meanwhile, Connell (1968) described diagnostic criteria based on the combination of symptoms such as abdominal pain and bowel function abnormalities without pathological changes [33]. Ritchie (1970) added the sensitivity of the colon while being pressured, as well as the profuse excretion and transition of the mucus to the list of typical symptoms [21]. Both researchers stressed the significance of excluding other gastroenterological diseases that might present with similar manifestations [21,33]. Moreover, Manousos et al. (1967) analyzed intestinal content transition time in 75 individuals with irritable colon syndrome, 43 patients with diverticulosis, and 88 subjects who had no abnormalities in bowel functions. The results showed that people suffering from both irritable colon and diverticulosis had a shortened transition time of food through the digestive tract. The researchers believed it might be related to a disturbance in colonic muscle function [34]. However, Cann et al. (1983) noted changed food transition time not only in the colon but also in the small intestine. It proved that IBS should be considered a disease affecting the whole intestine. The researchers also described that individuals with constipation had a prolonged transition time of intestinal content, whereas diarrheic ones shortened [35]. 

Since 1978, researchers and physicians have referred to the criteria described by Manning et al. (1978). They included flatulence, pain relief after intestinal movement, frequent and looser stools, the presence of mucus, or the impression of incomplete defecation as a highly possible differentiation of IBS from organic diseases [36]. A few years later, Kruis drew attention to the duration of symptoms as an essential diagnostic criteria, which was not universally accepted [37]. Subsequently, the Rome criteria for IBS were first presented at the 13th Rome Congress in 1988 and published as a result of Thomson, Drossman, Heaton, Dotteval, and Kruis collaboration [38]. The Rome Committee continued its work on the proper definition and diagnosis of IBS, which was amended in 1992, 1999, and 2006. The latest version was presented in 2016 as the Rome IV criteria. It states that IBS must manifest with abdominal pain relapsing at least one day per week for the past three months, began at least six months before diagnosis, and correlate with no less than two of the following criteria: being linked to defecation, be associated with shifts in stool regularity and/or its form [37]. Besides the typical symptoms of IBS, nausea, vomiting, heartburn, or even anxiety, insomnia, or depression have been observed in some patients suffering from the disease [39]. 

As more became known about IBS, more attention was paid to the treatment methods. In 1966, doctor MacDougall recommended a psychological and physical approach to managing the disease. This included reducing bowel stimulation through a diet adapted to the observed symptoms, the use of pharmaceuticals (e.g., codeine phosphate, diphenoxylate, propantheline), and reducing stress [40]. Later, in 1977, Diamant published a review on irritable colon syndrome in which he analyzed the latest reports on the treatments of the disease. The researcher drew attention to the importance of psychological factors since almost 50% of patients responded positively to placebo in various trials. However, Dotevall and Groll (1974), in their studies on mepiprazole, a type of tranquilizer, found that the placebo was ineffective after some time compared to the analyzed substance. In addition to tranquilizers, antispasmodic drugs such as anticholinergics or mebeverine, as well as agents to increase stool mass, were also prescribed by physicians for patients with IBS, either individually or as a combination of treatments [41]. Diamant (1977) also suggested that a low-fiber diet may be responsible for the development of the syndrome. The researcher proposed the adjustment of the dosage of dietary fibers to the observed symptoms of patients as an approach to treat the disease [42]. Nowadays, the European Food Safety Authority (EFSA) defines dietary fibers as non-digestible carbohydrates, such as pectins, cellulose, resistant starch, and non-starch polysaccharides, or fructooligosaccharides, as well as lignin [43]. At the end of the 20th century, psychological treatments (e.g., psychotherapy, behavioral or group therapy, and hypnotherapy) and the exclusion of certain foods, were also acknowledged as an additional or alternative approach to the medical treatment of IBS [44]. Simultaneously, preliminary studies of agents modifying 5-HT receptors’ functionality had begun [45]. To date, the greatest interest in the pharmacological management of IBS patients is focused on receptor subtypes 5-HT_3_ and 5-HT_4_ due to their impact on the functionality of GIT [46]. In the early 2000s, it was hypothesized that 5-HT_3_ receptor antagonists such as ondansetron and granisetron might be effective in IBS treatment, while cilansetron was already approved for use [47]. Moreover, studies have been conducted on 5-HT_4_ receptor agonists, namely, tegaserod or prucalopride [48]. Currently, new 5-HT_3_ receptor antagonists and 5-HT_4_ receptor agonists are still being sought [49,50].

Since the late 1970s, researchers have searched for natural ways of symptomatic treatments of IBS because of the side effects associated with prolonged pharmaceutical treatment. One of the alternatives was peppermint oil, whose antispasmodic properties were observed both in vitro and in vivo by Rees et al. (1979) [51]. However, it was not until the turn of the 20th to the 21st century that probiotics began to be investigated as a means of treating IBS [28]. Furthermore, Hunter et al. (1999) initiated studies on prebiotics as management of IBS [52]. Nowadays, synbiotics are also a subject of interest in IBS management analysis. However, even after 2010, there was not much data available on synbiotics’ impact on gastrointestinal disorders [53,54]. To date, research groups have focused on probiotics, prebiotics, and synbiotics as means to help IBS patients.

## 4. Intestinal Microbiota in Patients with Irritable Bowel Syndrome

Human GIT is colonized by up to 10^14^ organisms belonging to about 1000 species, among which prokaryotes (bacteria and unicellular microbes) dominate. However, fungi, archaea, parasites, and viruses are also inhabitants of the gut [55,56]. Bacteria residing in the GIT are mostly classified into four phyla, namely, *Firmicutes*, *Bacteroidetes*, *Proteobacteria*, and *Actinobacteria* [57]. Differences in the GIT microbiota of individuals can be attributed to the type of delivery and feeding of the infant, age, sex, diet, sanitary and living conditions, health issues, and administrated pharmaceuticals, as well as geographical regions [58]. Nevertheless, dysbiosis of the GIT microbiota can trigger immune system responses that result in inflammation in the gut and disruption of the GBA [59]. The imbalance of the gut microbiota, which may be associated with overgrowth or lack of certain microorganisms, or genetic abnormalities, can lead to a variety of cardiovascular, neurological, or intestinal diseases, one of which is IBS [19,57]. 

Pittayanon et al. (2019) conducted a systematic review of 24 studies on the microbiota of IBS patients and observed significant differences in the results. These researchers found that in some studies, the number of potentially harmful bacteria from *Enterobacteriaceae* family and *Bacteroides* genus was increased. Simultaneously, a decrease in the prevalence of representatives of the beneficial microbiota, namely, genus *Bifidobacterium* and *Faecalibacterium*, was observed in individuals with IBS compared to the healthy ones [60]. Chong et al. (2019) made analog observations in their review. Additionally, the article mentioned the increased number of bacteria belonging to *Firmicutes* phyla, including *Lactobacillus* and *Ruminococcus* genus, as well as a decreased abundance of *Erysipelotrichaceae* and methanogens in IBS patients [61]. Mei et al. (2021), who analyzed only IBS-D patients in China, also observed elevated abundance of bacteria belonging to *Enterobacteriaceae* family, as well as Proteobacteria phyla, and decreased prevalence of *Firmicutes*, *Fusobacteria* phyla, and *Alloprevotella*, Fusobacterium genus in contrast to the healthy population [62]. Tap et al. (2017) observed the correlation between the severity of IBS and the number of *Methanobacteriales* capable of producing methane, which is linked to the occurrence of constipation [63]. In addition, the research group noted the reduced number of bacteria from *Prevotella* genus in IBS patients compared to healthy subjects. However, Su et al. (2018) and Barandouzi et al. (2021) found increased abundance of this genus in IBS individuals [63,64,65]. What is more, Shukla et al. (2015) found a lower abundance of *Bifidobacterium* and *Lactobacillus* genus and increased prevalence of *Veillonella* genus, *Clostridium coccoides*, *Bacteroides thetaiotamicron*, *Ruminococcus productus*, and *P. aeruginosa* [66]. Although several studies have described an increased ratio of *Firmicutes* to *Bacteroidetes* in patients with IBS compared to healthy individuals, Barandouzi et al. (2021) observed a similar abundance of bacteria belonging to these two phyla, that constitute about 90% of the total microbiota of GIT [65,67]. This research group observed, among IBS patients, a higher prevalence of bacteria belonging to *Verrucomicrobia* phyla, as well as from *Blautia* genus, which is acknowledged as a marker of microbiota imbalance [65]. Similarly, Lee et al. (2021) found no significant differences in phyla levels in fecal samples of IBS patients and healthy population. However, these researchers observed an increase in pathogenic bacteria from *Desulfovibrionaceae* family, and a simultaneous decrease in the beneficial *Lachnospiraceae* family [68]. On the contrary, Ahluwalia et al. (2021) did not notice any differences between the fecal microbiota of IBS and healthy individuals. They concluded the variations were likely related to microbiota functionality rather than composition [69]. Moreover, Dlugosz et al. (2015) reported no significant differences in the small intestine microbiota of IBS and healthy subjects [70]. 

Despite the efforts of researchers to find the dysbiosis patterns in the microbiota of people suffering from IBS, there are still many inconsistencies in the obtained results. It could be due to different GIT parts from which samples were gathered, different analytical methods, or even population disparity [71].

## 5. Probiotics, Prebiotics, and Synbiotics in IBS Treatment

Growing evidence of GIT microbial population disturbances and gastroenteritis being factors of IBS development resulted in the search for therapies based on microbiota manipulations. For this purpose, probiotics, prebiotics, and synbiotics could be used [72,73]. To date, researchers have carried out multiple studies on the impact of various probiotic strains, prebiotics, and their mixtures on people suffering from IBS. A few studies were conducted on animal models which can be applied if the etiology of the induced disease is as similar as possible as it is in humans. These models play an important role in pre-clinical research on the treatment or mechanisms of functional gastrointestinal disorders, including IBS [74]. Since psychological pressure might cause the development of IBS or provoke its symptoms in humans, stress is an inducing factor for most animal models. Nevertheless, IBS might emerge in individuals after infections; therefore, the post-infectious animal models are also used, which are caused by pathogenic bacteria or parasites. Chemical or mechanical stimulation might also trigger IBS symptoms in rodents [75].

### 5.1. Probiotics

The probiotic effect is attributed to the strain and even two different strains of the same species might impact the patient to various extension [76,77]. Therefore, the influence of one probiotic cannot be extrapolated to another one from the same species or even to a different strain [78]. Moreover, probiotics can act differently in various populations, as well as stages and types of diseases [77,78].

In 2002, Sen et al. published a study on the influence of *Lactobacillus plantarum* 299V on IBS patients. The strain did not exhibit any beneficial effect on the disease symptoms [79]. On the contrary, Ducrotté et al. (2012) described the potentially beneficial impact of the strain on the IBS symptoms of studied subjects [80]. Nevertheless, the research conducted by Sen et al. (2002) was not the only one leading to the conclusion that tested probiotic might not be effective in IBS treatment [79]. Pedersen et al. (2010), Simrén et al. (2010), Søndergaard et al. (2011), Amirimani et al. (2013), Roberts et al. (2013), Lorenzo-Zúñiga et al. (2014), and Cremon et al. (2018) obtained analog results [81,82,83,84,85,86,87]. Both Simrén et al. (2010), and Søndergaard et al. (2011) analyzed probiotic Cultura, which was milk fermented with *Lactobacillus bulgaricus*, as well as *Streptococcus thermophilus*, and containing three probiotic strains, namely, *Lactobacillus paracasei* F19, *Lactobacillus acidophilus* La5, and *Bifidobacterium animalis* subsp. *lactis* Bb12 [82,83]. Pedersen et al. (2010), who studied the same probiotic fermented milk product, observed that acidified milk itself caused the improvement in IBS symptoms [81]. Robert et al. (2013) also studied dairy product, which was yogurt with starter cultures of *S. thermophilus* CNCM I-1630, *Lb. bulgaricus* CNCM I-1632 and *Lb. bulgaricus* CNCM I-1519, as well as probiotic strain *B. lactis* DN-173 010 [85]. On the other hand, Amirimani et al. (2013) analyzed the influence of probiotic tablets Biogaia^®^ containing *Lactobacillus reuteri* on IBS patients [84]. Lorenzo-Zúñiga et al. (2014) conducted research on encapsulated multi-strain probiotic, including *Lb. plantarum* CECT7484, *Lb. plantarum* CECT7485, and *Pediococcus acidilactici* CECT7483, whereas Cremon et al. (2018) analyzed the influence of capsules containing *Lb. paracasei* CNCM I-1572 [86,87]. Furthermore, Ligaarden et al. (2010) observed an unfavorable effect of encapsulated *Lb. plantarum* MF1298 on IBS subjects [88].

However, there are several trials, which proved the beneficial influence of probiotics on IBS symptoms and their severity. Among them, there were probiotic capsules with *Bifidobacterium infantis* 35,624, or *Bifidobacterium bifidum* MIMBb75, or *Lactobacillus brevis* KB290 studied by Whorwell et al. (2006), Guglielmetti et al. (2011), and Murakami et al. (2012), respectively [89,90,91]. Additionally, Martoni et al. (2020) performed the analysis involving two separate probiotics *Lb. acidophilus* DDS^®^-1, and *B. lactis* UABla-12™, in a form of capsules. Besides improvement in overall IBS symptoms, Martoni et al. (2020) described the positive influence of both probiotic strains on stool consistency and the severity of abdominal pain. Additionally, *Lb. acidophilus* DDS^®^-1 contributed to the reduction of stress levels of IBS individuals [92]. Caviglia et al. (2020) and Zhou et al. (2020) used *Bifidobacterium langum* as a probiotic in studies conducted on humans and rats, respectively [93,94]. Caviglia et al. (2020), who analyzed strain *B. langum* ES1, observed improvement in overall IBS symptoms and enhanced intestinal barrier integrity, as well as immune-inflammatory state of IBS-D patients [93]. Similarly, Zhou et al. (2020) noted the improvement of intestinal permeability, along with positive changes in GIT microbiota in the rat WAS (water avoidance stress) model after *B. langum* treatment [94]. However, contrary to Caviglia et al. (2020), the research team did not establish any impact of *B. langum* on serum cytokines levels in rats [93,94]. Lewis et al. (2020) conducted a trial involving two separate probiotics. Besides *B. longum* HA-196 *Lb. paracasei* R0175 was administrated to IBS individuals. Both probiotics ameliorated the social quality of participants’ lives. Although, only *B. langum* HA-196 contributed to the increase of *Bifidobacterium* genus abundance in fecal samples, which was not observed for *Lb. paracasei* R0175 and *corresponding Lactobacillus* spp. [95]. What is more, *Lb. gasseri* BNR17 has been described by Kim et al. (2018) as suitable for use as IBS treatment since it improved its symptom [96]. Shin et al. (2018) analyzed the same strain and noted its impact on intestinal microbiota, as well as bowel habits of trial participants [97]. *Lb. casei* is yet another species from *Lactobacillus* genus, which researchers studied as probiotics in IBS treatment [98,99]. Compare et al. (2017), who performed ex vivo analysis on ileal and colonic mucosa culture tissue model harvested from PI-IBS-D patients, established that *Lb. casei* DG is able to diminish mucosal inflammation [98]. On the other hand, Seong et al. (2021) conducted a study on a rat IBS model induced with chronic restrain stress. Those researchers also observed the capability of *Lb. casei* DKGF7 to decrease inflammatory cytokines in colonic tissue, as well as serum corticosterone levels, along with amelioration of IBS symptoms and increased expression of tight junction proteins [99]. Furthermore, Dapoigny et al. (2012) described the trial in which participants received *Lb. rhamnosus*. This probiotic improved symptoms of IBS only among individuals suffering from the IBS-D subtype [100]. Other effective probiotics in improving IBS symptoms are strains of *Bacillus coagulans* [101,102,103,104]. Majeed et al. (2018) observed that *B. coagulans* MTCC 5856 could reduce sleeplessness and depression in IBS-D patients [102]. Gupta et al. (2012) noted the beneficial impact of *B. coagulans* LBSC on GIT microbiota of IBS individuals [104]. Likewise, Sun et al. (2018) used *Clostridium butyricum* as IBS treatment in the trial, in which probiotic exhibited the ability to ameliorate symptoms of the disease. However, no significant impact on the intestinal microbiota of tested subjects was observed [105]. Zhao et al. (2019) introduced *C. butyricum* probiotic strain to mice PI-IBS model induced with 2,4,6-trinitrobenzenesulfonic acid (TNBS). They described a decrease in intestinal visceral hypersensitivity, along with reduced low-grade mucosal inflammation [106]. Interestingly, only Kruis et al. (2012) used *Escherichia coli* Nissle 1917 (MUTAFLOR^®^) in their trial on IBS-C and IBS-D patients and concluded that it poses therapeutic potential for PI-IBS, as well as post-antibiotic IBS individuals [107]. Other rarely studied probiotics are *Saccharomyces* spp. yeast, among which *Saccharomyces cerevisiae* CNCM I-3856 strain was used as probiotic in de Chambrun et al. (2015) and Spiller et al. (2016) studies. De Chambrun et al. (2015) observed reduced severity of abdominal pain and discomfort, whereas Spiller et al. (2016) noted overall improvement of IBS symptoms but only among IBS-C subjects [108,109]. Moreover, Abbas et al. (2014) described the potential of *Saccharomyces boulardii* to reduce inflammation in the GIT of IBS-D individuals [110]. Probiotic *S. boulardii* yeast were also used as a component of multi-strain preparations studied by Hong et al. (2019), as well as Leventogiannis et al. (2019) [111,112]. Hong et al. (2019), who used the PI-IBS mice model, established that studied probiotic preparation composed of *S. boulardii*, *Lb. acidophilus* LA-5, and *B. lactis* BB-12, contributed to the reduction of serum levels of pro-inflammatory cytokines, and visceral hypersensitivity [111]. Leventogiannis et al. (2019) conducted human studies and noted that Lactolevure^®^ probiotic, including *S. boulardii*, *B. lactis* BB-12, *Lb. acidophilus* LA-5, and *Lb. plantarum*, can be effective in attenuation of bloating and abdominal pain severity, especially in patients with IBS combined with small intestinal bacterial overgrowth (SIBO) [112]. Reduction of SIBO was also observed by Barret et al. (2008), who studied Yakult^®^ dairy product containing *Lactobacillus casei* Shirota. This probiotic also decreased the early rise in breath hydrogen after lactulose (ERBHAL), and, simultaneously, abdominal pain [113]. Lee et al. (2018) described the comparable observation concerning the influence of probiotic on SIBO. Their trial involved only IBS-D patients treated with a multi-strain probiotic (Ther-Biotic^®^ Complete) containing *S. thermophilus*, along with strains belonging to *Lactobacillus* spp. (7) and *Bifidobacterium* spp. (4) [114].

Multi-strain probiotic preparations are widely studied as the mode of IBS management [81,82,83,85,86,111,112,114,115,116,117,118,119,120,121,122,123,124,125,126]. They can be effective in attenuation of IBS symptoms, such as, ones including *Lb. acidophilus* SDC 2012, along with *Lb. acidophilus* SDC 2013 tested by Sinn et al. (2008), as well as a bifid triple viable capsule containing *B. longum*, and *Lb. acidophilus* used in Cui and Hu (2012) trial, or Bio-Kult^®^ composed of five *Bifidobacterium* spp. and six *Lactobacillus* spp. strains, along with *S. thermophilus* PXN 66 analyzed by Ishaque et al. (2018) [115,118,123]. Sisson et al. (2014) described another multi-strain probiotic Symprove, comprising *Lb. rhamnosus* NCIMB 30174, *Lb. plantarum* NCIMB 30173, *Lb. acidophilus* NCIMB 30175, and *Enterococcus faecium* NCIMB 30176, which exhibited a beneficial impact on IBS symptoms [119]. Oh et al. (2019), also described the amelioration of IBS symptoms among participants of the trial, which excluded individuals suffering from IBS-C, after treatment with Foodies Lactobacillus (*Lb. paracasei*, *Lb. salivarius*, *Lb. plantarum*) [124]. The results of this study indicate the ability of the preparation to reduce the severity of abdominal pain, which was similar to results obtained by Hong et al. (2009), Zhang et al. (2019), and Skrzydło-Radomańska et al. (2021) [116,124,125,126]. Hong et al. (2009) used the preparation including *B. bifidum* BGN4, *B. lactis* AD011, *Lb. acidophilus* AD031, and *Lb. casei* IBS041 [116]. Bifico^®^ (*B. longum*, *Lb. acidophilus*, *E. faecalis*) administrated to IBS-D individuals in the trial conducted by Zhang et al. (2019) not only reduced abdominal pain but also exhibited beneficial impact on GIT microbiota and short-chain fatty acids (SCFAs) concentrations, as well as reduced plasma levels of cytokines [125]. Skrzydło-Radomańska et al. (2021) observed additional improvement in quality of life after treatment of IBS-D patients with NordBiotic™ including *S. thermophilus* ST250, as well as *Lactobacillus* spp. (5), and *Bifidobacterium* spp. (3) strains [126]. Michail and Kenche (2011), as well as Hod et al. (2018), focused their research on the influence of multi-strain probiotics on IBS-D patients’ microbiota [117,122]. Michail and Kenche (2011) did not observe any impact of VSL#3 probiotic (*S. thermophilus*, *B. breve*, *B. longum*, *B. infantis*, *Lb acidophilus*, *Lb plantarum*, *Lb paracasei*, *Lb. bulgaricus*) on fecal microbiota, and, additionally, no significant changes in participants’ body mass index (BMI) [117]. Additionally, Hod et al. (2018), who analyzed BIO-25, composed of *S. thermophilus* ST3, *Lactococcus lactis* SL6, along with *Lactobacillus* spp. (6), and *Bifidobacterium* spp. (4) strains, in management of IBS-D, noted that the preparation did not affect microbial diversity in participants’ feces. Nevertheless, they observed increased abundance of *Lactobacillus* spp. and *Lactococcus* spp. in stool samples of subjects whose abdominal pain and bloating were reduced, and a simultaneous decrease in *Bilophila* genus prevalence among individuals with decreased abdominal pain and improved stool consistency [122]. Furthermore, Yoon et al. (2015), established that LacClean Gold-S^®^ (*B. bifidum* KCTC 12199BP, *B. lactis* KCTC11904BP, *B. longum* KCTC 12200BP, *Lb. acidophilus* KCTC 11906BP, *Lb. rhamnosus* KCTC 12202BP, *S. thermophilus* KCTC 11870BP) improve symptoms only among patients with IBS-D, whereas probiotic strain included in the preparation were present in fecal samples of all trial participants [120]. Comparable results, on two different products containing *Lb. acidophilus* DSM 24936, and *Lb. reuteri* DSM 25175, or *Lb. rhamnosus* DSM 25568, *Lb. plantarum* DSM 24937, and *B. lactis* DSM 25566, were obtained by Mezzasalma et al. (2016) among individuals suffering from IBS-C [121].

Most of the cited research was carried out in double-blind mode; however, there are still some trial designs conducted without placebo, or control group [93,112,113,114,125], as well as research conducted in full awareness of investigators, and/or participants of the administrated product (treatment/control) [81,93,112,113,114,125]. Moreover, the number of studies including the dose-related effect of probiotics on IBS subjects is very limited [86,89,96]. Animal or ex vivo culture tissue models are also rarely used for the analysis of active agents’ potential impact on IBS individuals [94,98,99,111]. Most of the above-described studies are focused on all IBS subtypes [80,81,82,87,88,89,95,100,108,109,112,113,115,116,118,119,120]. Although, several studies did not differentiate the form of the disease among participants [79,83,84,85,90,91,92,96,101,103,104,127]. Moreover, Oh et al. (2019) performed the analysis of probiotic impact on IBS patients with the exclusion of subjects suffering from IBS-C [124]. This subtype was chosen as the inclusion criteria only by Kruis et al. (2012) and Mezzasalma et al. (2016) [107,121]. However, Kruis et al. (2012) included also people with IBS-D, which was the subtype on which Michail and Kenche (2011), Abbas et al. (2014), Lorenzo-Zúñiga et al. (2014), Majeed et al. (2016), Compare et al. (2017), Hod et al. (2018), Ishaque et al. (2018), Lee et al. (2018), Shin et al. (2018), Sun et al. (2018), Zhang et al. (2019), Caviglia et al. (2020), and Skrzydło-Radomańska et al. (2021) focused [86,93,97,98,102,105,107,110,114,117,122,123,125,126]. Details of the above-mentioned trials are listed in Table 1.

### 5.2. Prebiotics

At present, only a few pieces of research on the impact of prebiotics on the health improvement of people suffering from IBS have been conducted. Therefore, this area of study is still in need of investigation.

The first study on the influence of prebiotics on IBS management was published in 1999 by Hunter et al., who analyzed the oligofructose, the effect of which was marginal and only in patients with IBS-C [52]. Similarly, Olesen et al. (2000), who studied fructooligosaccharides (FOS), did not conclude whether use of prebiotic helped to improve the condition of IBS patients or not [128]. Short-chain FOS (scFOS) was also studied by Azpiroz et al. (2016), who described the influence of the prebiotic on the anxiety level of IBS individuals and *Bifidobacterium* spp. count in their stool [129]. Silk et al. (2009) tested the effectiveness of another prebiotic, namely, the trans-galactooligosaccharide (GOS) mixture produced by *Bifidobacterium bifidum* NCIMB 41171 from lactose. They observed that it not only relieved symptoms such as flatulence, abdominal pain, and discomfort, as well as stool patterns but also increased the number of *Bifidobacterium* spp. in fecal samples [130]. Both GOS and FOS, along with inulin and anthocyanins, were included in the preparation used in the trial conducted by Chen et al. (2017) in the IBS mice model. The product exhibited the ability to diminish inflammation and improve the intestinal barrier. Additionally, if used prior to infection, it could help to establish PI microbial homeostasis in the GIT. Nonetheless, the blend of prebiotics needs to be further investigated in humans [131]. Niv et al. (2016) proved the effectiveness of partially hydrolyzed guar gum (PHGG) for IBS patients suffering mostly from gasses and bloating. The use of this compound did not cause any side effects. However, it did not exhibit any influence on the rest of the possible IBS symptoms [132].

All of the cited research was placebo-controlled [52,128,129,130,132], except from animal studies described by Chen et al. (2017) [131]. Among mentioned human trials, only ones conducted by Olesen et al. (2000) [128], and Silk et al. (2009) [130], were not carried out in a double-blind system. However, the study design by Silk et al. (2009) was the only one including the influence of a dose on prebiotic effectiveness in IBS therapy [130]. Research carried out by Hunter et al. (1999) and Olesen et al. (2000) did not differentiate IBS subtypes [52,128], whereas ones conducted by Silk et al. (2009), Azpiroz et al. (2016), and Niv et al. (2016) included patients exhibiting all forms of the disease [129,130,132]. Details of the above-mentioned research are presented in Table 2.

### 5.3. Synbiotics

Despite the concept of synbiotics being introduced in 1995, and the first attempts to recognize IBS were made at the end of the 19th century, the idea of using these preparations to treat the disease appeared in the last decade [133].

In 2013, Cappello et al. performed the first analysis of Probinul^®^ and its impact on IBS individuals. The synbiotic included inulin, tapioca-resistant starch, and *Lactobacillus* spp. (6) and *Bifidobacterium* spp. (2) strains, as well as *Streptococcus thermophilus*. Probinul^®^ did not diminish flatulence and bloating to the satisfying level according to participants of the trial [134]. Shavakhi et al. (2014), who studied the impact of Balance^®^, which included FOS, and probiotic strains from *Lactobacillus* (4) and *Bifidobacterium* (3) genus, as well as *S. thermophilus*, did not observe any influence of the preparation on IBS patients [135]. Similar results were obtained by Bogovič Matijašic et al. (2016), who studied synbiotic fermented milk product containing *Lb. acidophilus* La-5, *B. lactis* BB-12, and 2% dietary fiber (Beneo Orafti Synergy1; 90% inulin, 10% oligofructose) [136]. On the contrary, Bucci et al. (2014) noted the attenuation of flatulence in IBS subjects after 4 weeks of the disease treatment with Probinul^®^, which was sustained during a 6 months period of therapy [137]. Another synbiotic, namely, Lactol^®^, including *B. coagulans* and FOS, was tested by Rogha et al. (2014). The research team noted relief of abdominal pain, discomfort, and diarrhea in IBS individuals; however, no improvement in constipation-related symptoms was observed. Nonetheless, the study revealed some side effects of the preparation; therefore, its safety has to be further evaluated [54]. Although, Asgarshirazi et al. (2015), who analyzed the effectiveness of Lactol^®^ in treating functional abdominal pain in children, did not notice any side effects of the preparation [138]. Moser et al. (2019) conducted a study on the impact of yet another synbiotic preparation named OMNi-BiOTiC^®^ Stress Repair on IBS-D patients. The preparation comprised prebiotics, namely, corn starch, maltodextrin, inulin, and FOS, along with *Lactococcus lactis* W19, *Lactobacillus* spp. (5), and *Bifidobacterium* spp. (3) strains. Results showed a positive influence of the mixture on mucosal microbiota diversity and concentrations of acetate and butyrate in fecal samples [139]. Lee et al. (2019) studied the impact of another synbiotic preparation, containing inulin, FOS, and probiotic strains from *Lactobacillus* (6) and *Bifidobacterium* (2) genus on IBS patients. The research team observed improvement in bloating, fatigue, and abdominal discomfort in trial participants [140]. On the other hand, Min et al. (2012) and Bahrudin et al. (2020) analyzed the influence of synbiotic dairy products, such as yogurt with the addition of *Bifidobacterium animalis* *subsp. lactis* Bb-12 and acacia dietary fiber, and drink containing *Lb. helveticus* and polydextrose as a prebiotic, respectively, on IBS subjects [141,142]. Min et al. (2012) described that the studied product could attenuate symptoms of the disease in both IBS-C and IBS-D patients [141]. Bahrudin et al. (2020) observed the beneficial effect of synbiotic only in studied IBS-C individuals. Nevertheless, the researchers concluded that probiotic strain alone is the active agent [142]. The effective mixture of pro- and prebiotics for IBS-D patients, which reduce the feeling of incomplete intestinal movements, release abdominal pain, and help to regulate stool patterns, have been described by Skrzydło-Radomańska et al. (2021). The synbiotic comprised probiotics, namely, *Lb. rhamnosus* FloraActive 19070-2, *Lb. acidophilus* DSMZ 32418, *B. lactis* DSMZ 32269, *B. longum* DSMZ 32946, *B. bifidum* DSMZ 32403 strains, and scFOS as a prebiotic compound [143]. Last but not least, in 2020, Seong et al. described a synbiotic, containing *Lb. paracasei* DKGF and *Opuntia humifusa* extract as a prebiotic, effective in IBS murine model. Nevertheless, its functionality must be further assessed in humans [144]. 

Most of the cited research [54,134,135,136,137,140,142,143] was conducted as double-blind, placebo-controlled, except from Moser et al. (2019) [139], as well as Seong et al. (2020) [144], who performed the animal study. Two studies were conducted on people suffering from the IBS-C subtype [136,142], and the other two on subjects with IBS-D [139,143]. Four studies did not differentiate subtypes of the disease [54,134,135,137], whereas the rest of mentioned research focused on all subtypes [140,141]. Furthermore, only one trial involved dose-related dependencies [140]. Detailed descriptions of the aforementioned studies are listed in Table 3.

## 6. Summary

In conclusion, the use of probiotics, prebiotics, and synbiotics in IBS treatment is still in need of investigation and standardization. Researchers focused mostly on probiotics, especially multi-strain ones, creating a demand for new research on prebiotics, and synbiotics in the management of IBS.

The vast majority of trials are double-blind and placebo-controlled; however, there are still studies conducted in an open-label system, as well as with no control or placebo group, which could bring unreliable results. Additionally, analyzed preparations differ in probiotic strains, their number, and density, as well as amount and type of prebiotic or their combination, in the case of synbiotics. Moreover, the dosage of studied preparations varied among research, and its impact is rarely analyzed. Most of the trial designs would benefit from a longer treatment period and additional follow-up phase since this aspect is mostly described by researchers as a study limitation. Another obstruction of research might be the number of participants that rarely exceeded 100. However, this obstacle is hard to avoid because individuals are entering the trials voluntarily. Lastly, not every probiotic, prebiotic, and their combination would be an appropriate mode of treatment for each IBS subtype, which also needs to be studied in more detail.

Nevertheless, the last two decades of research on probiotics, prebiotics, and synbiotics bring satisfying results and they are acknowledged as effective and safe in IBS therapy. These preparations can be introduced as an alternative to drugs that might carry a risk of side effects, especially in long-term use. Among the field of microbiota-manipulation-based therapies, probiotics, prebiotics, and synbiotics are a promising direction of alleviation of symptoms for people suffering from IBS.

## Figures and Tables

**Table 1:** Studies on the impact of probiotics on IBS patients.

Paper	Research Type	Participants Selection Criteria	Participants ^1^	IBS Subtype	Preparation (Dosage)	Placebo/Control (Dosage)	Duration	Outcomes
Sen et al. (2002) [79]	The randomized, double-blind, placebo-controlled study	Rome I	12 males/females 18–65 y ^2^	wd ^3^	ProViva (oatmeal gruel, 5 × 10^7^ cfu/mL ^4^ of *Lb. plantarum* 299V) 125 mL/d ^5^	Oatmeal gruel 125 mL/d	First 4 weeks of placebo administration, then 4 weeks of treatment	No beneficial effect
Whorwell et al. (2006) [89]	The randomized, double-blind, placebo-controlled, multicenter, dose-ranging stud	Rome II	362 females 18–65 y	All	Excipient (no data on used compounds), *B. infantis* 35,624 (three different doses: 1.0 × 10^6^, 1.0 × 10^8^, or 1.0 × 10^10^ cells/capsules) 1 capsule/d ^6^	Excipient (no data on used compounds) 1 capsule/d	2 weeks of the run-in period, then 4 weeks of treatment, and 2 weeks of follow up	Improvement of IBS symptoms The effective dose was 1.0 × 10^8^ cells/capsule
Barrett et al. (2008) [113]	Uncontrolled pilot study	Rome II	18 males/females 20–70 y	All	Yakult^®^ (sucrose, skim milk powder, dextrose, 6.5 × 10^9^ cells/dose *L. casei* Shirota) 65 mL/d	na ^7^	Up to 2-week run-in period, 6 weeks of treatment	Reduction in SIBO ^8^ Regression of ERBHAL ^9^, accompanied by improved abdominal symptoms
Sinn et al. (2008) [115]	The randomized, double-blind, placebo-controlled human study	Rome III	40 males/females 18–70 y	All	Excipient (no data on used compounds), freeze-dried *Lb. acidophilus* SDC 2012, and *Lb. acidophilus* SDC 2013 (a total of 2.0 × 10^9^ cfu/mL) 2 capsules/d	Excipient (no data on used compounds) 2 capsules/d	4 weeks	Improved IBS symptoms
Hong et al. (2009) [116]	The randomized, double-blinded, placebo-controlled parallel-group clinical study	Rome III	70 males/females 19–75 y	All	*B. bifidum* BGN4, *B. lactis* AD011, *Lb. acidophilus* AD031, *Lb. casei* IBS041 (a total of 2.0 × 10^10^ cfu/packet ^10^ viable, lyophilized probiotics) 2 packets/d ^11^	Skim milk powder 2 packets/d	8 weeks	Reduced abdominal pain and defecation discomfort
Ligaarden et al. (2010) [88]	The randomized double-blind, placebo-controlled, crossover trial	Rome II	16 males/females 18–75 y	All	10^10^ cfu/capsule ^12^ *Lb. plantarum* MF1298 (live, freeze-dried) 1 capsule/d	No data on used compounds 1 capsule/d	1 week run-in period, followed by 3 weeks of treatment (probiotc/placebo), then 4 weeks of wash-out phase, and another 3 weeks of alternate treatment (placebo/probiotic)	Adverse effects of probiotic
Pedersen et al. (2010) [81]	Placebo-controlled study	Rome II	61 males/females 18–79 y	All	Milk (a total of 10^7^–10^9^ cfu/g ^13^ acidifiers: *Lb. delbruckeii* ssps. *bulgaricus*, *S. thermophilus*; probiotics: 5 × 10^7^ cfu/mL *Lb. paracasei* F19, 5 × 10^7^ cfu/mL *Lb acidophilus* LA-5, 5 × 10^7^ cfu/mL *B. lactis* BB-12) 400 mL/d	Milk (acidifiers: D-(+)-gluconic acid, δ-lactone) 400 mL/d	2 weeks of wash-out period, then 8 weeks of treatment	Observed effects of treatment were due to acidified milk itself, not the probiotic
Simrén et al. (2010) [82]	The randomized, double-blind, placebo-controlled study	Rome II	74 males/females 18–70 y	All	Cultura (fermented with: *Lb. bulgaricus*, *S. thermophilus*; containing probiotics: 5.0 × 10^7^ cfu/mL *Lb. paracasei* F19, 5.0 × 10^7^ cfu/mL *Lb. acidophilus* La5, 5.0 × 10^7^ cfu/mL *B. lactis* Bb12) 400 mL/d	Acidified milk 400 mL/d	2 weeks of the run-in period, then 8 weeks of treatment, and 8 weeks of follow-up	No beneficial effect
Guglielmetti et al. (2011) [90]	The prospective, multicenter, randomized, double-blind, placebo-controlled, two-arm nutritional study	Rome III	122 males/females 18–68 y	wd	Excipient (no data on used compounds), 1 × 10^9^ cfu/capsule *B. bifidum* MIMBb75 1 capsule/d	Excipient (no data on used compounds), maltodextrin 1 capsule/d	2 weeks of the run-in period, then 4 weeks of treatment, and 2 weeks of wash-out phase	Improvement of IBS symptoms Maintained beneficial impact of probiotic during the wash-out period
Michail and Kenche (2011) [117]	The randomized, double-blind, placebo-controlled trial	Rome III	24 males/females average age of 21.8 ± 17	IBS-D	VSL#3 (cornstarch, *S. thermophilus*, *B. breve*, *B. longum*, *B. infantis*, *Lb acidophilus*, *Lb plantarum*, *Lb paracasei*, *Lb. bulgaricus*) 9.0 × 10^11^ cells/d ^14^	Cornstarch	8 weeks	No impact on fecal microbiota No influence on BMI
Søndergaard et al. (2011) [83]	The randomized, double-blind, placebo-controlled, parallel-group trial	Rome II	64 males/females 18–70 y	wd	Cultura 1000 mL/d	Acidified milk 1000 mL/d	2-week run-in period, then 8 weeks of treatment	No effect on IBS symptoms
Cui and Hu (2012) [118]	The double-blind, placebo-controlled study	Rome III	60 males/females average age between 44.38 ± 15.08 and 48.45 ± 14.08	All	Bifid triple viable capsule *(B. longum*, *Lb. acidophilus*) 6 capsules/d	No data on used compounds 600 mg/d ^15^	4 weeks	Improvement in overall IBS symptoms Higher abundance of gene copies of *Bifidobacterium* spp. and *Lactobacillus* spp. in feces
Dapoigny et al. (2012) [100]	The prospective, multicenter, randomized, double-blind, placebo-controlled, parallel-group pilot trial	Rome III	50 males/females 18–70 y	All	2 × 10^8^ cfu/capsule *Lb. rhamnosus* (total freeze-dried culture) 3 capsules/d	No data on used compounds 3 capsules/d	2 weeks of the run-in period, then 4 weeks of treatment, and 2-week follow-up	Improvement of symptoms only among IBS-D patients
Ducrotté et al. (2012) [80]	The multicenter, parallel-group, double-blind, placebo-controlled study	Rome III	216 males/females 18–70 y	All	Excipients (potato starch, magnesium stearate) and 1 × 10^10^ cfu/capsule *Lb. plantarum* 299V DSM 9843	Potato starch and magnesium stearate	4 weeks of treatment, then 3 weeks of follow-up	Potentially beneficial in the management of IBS
Kruis et al. (2012) [107]	The randomized, double-blind, parallel-group, monocenter study	Rome II	120 males/females 18–65	IBS-C, IBS-D	MUTAFLOR^®^ (2.5–25 × 10^9^ cfu/capsule *Escherichia coli* Nissle 1917) 1 capsule/d (first 4 days) 2 capsules/d (the rest of the trial)	No data on used compounds 1 capsule/d (first 4 days) 2 capsules/d (the rest of the trial)	12 weeks	Therapeutic potential for PI-IBS ^16^ PA-IBS ^17^ subjects
Murakami et al. (2012) [91]	The placebo-controlled, double-blind, crossover trial	Rome III	35 males/females ≥ 6 y	wd	Corn starch, maltitol syrup, hydroxypropyl methylcellulose, calcium stearate, ≥1.0 × 10^10^ cfu/capsule *Lb. brevis* KB290 1 capsule/d	Corn starch, maltitol syrup, hydroxypropyl methylcellulose, calcium stearate1 capsule/d	4-week run-in period, followed by 4 weeks of treatment (probiotic/placebo), then 4 weeks of wash-out phase, and another 4 weeks of alternate treatment (placebo/probiotic)	Increased abundance of *Bifidobacterium* spp. and decrease of *Clostridium* spp. in fecal samples Improvement of IBS symptoms and subjects’ quality of life
Amirimani et al. (2013) [84]	The randomized parallel-group, single-blind, placebo-controlled study	Rome III	72 subjects (no information on sex, and age)	wd	Biogaia^®^ (1 × 10^8^ viable cells of *Lb. reuteri*) 1 tablet/d ^18^	1 tablet/d	4 weeks	Increased frequency of defecation No vital differences in IBS symptoms
Roberts et al. (2013) [85]	The randomized, double-blind, placebo-controlled trial	Rome III	179 males/females 18–65 y	wd	Yogurt with 1.2 × 10^9^ cfu/cup ^19^ of standard strains (*S. thermophilus* CNCM I-1630, *Lb. bulgaricus* CNCM I-1632, and I-1519) with the addition of 1.25 × 10^10^ cfu/cap *B. lactis* DN-173 010 2 cups/d ^20^	Milk-based non-fermented dairy product without probiotics and with similar lactose content to the test product2 cups/d	12 weeks	No impact of tested probiotic on IBS symptoms severity
Abbas et al. (2014) [110]	The randomized, double-blind, placebo-controlled trial	Rome III	72 males/females 18–60 y	IBS-D	*S. boulardii* 750 mg/d	No data on used compounds 750 mg/d	2 weeks run-in period, then 6 weeks of treatment	Decreased blood levels of pro-inflammatory cytokines (IL ^21^-8, TNF-α ^22^) Increased tissue levels of anti-inflammatory cytokines (IL-10)
Lorenzo-Zúñiga et al. (2014) [86]	The multicenter, randomized, double-blind, placebo-controlled intervention clinical trial	Rome III	84 males/females 20–70 y	IBS-D	*Lb. plantarum* CECT7484, *Lb. plantarum* CECT7485, and *P. acidilactici* CECT7483 (ratio 1:1:1; a total of 1–3 × 10^10^ cfu/capsule in high dose or 3–6 × 10^9^ cfu/capsule in low dose) 1 capsule/d	No data on used compounds 1 capsule/d	6 weeks	Improvement of IBS-related life quality No significant relief in the severity of IBS symptoms Lack of significant improvement of IBS symptoms Amelioration of quality of subjects’ life
Sisson et al. (2014) [119]	The single-center, randomized, double-blind, placebo-controlled trial	Rome III	186 males/females 18–65 y	All	Symprove (water-based barley extract, *Lb. rhamnosus* NCIMB 30174, *Lb. plantarum* NCIMB 30173, *Lb. acidophilus* NCIMB 30175, *E. faecium* NCIMB 30176; total of 1.0 × 10^10^ bacteria/50 µ) 1 mL/kg a day ^23^	Water, flavorings 1 mL/kg a day	12 weeks of treatment, and 4 weeks of follow-up	Significantly improved symptoms of IBS, especially pain and bowel habits Ameliorated symptoms severity The effect was not sustained during the follow-up period
Urgesi et al. (2014) [101]	The monocentric, double-blind, placebo-controlled, parallel-group clinical trial	Rome III	52 males/females 18–75 y	wd	Colinox^®^ (excipient—no data on used compounds, simethicone, 1.5 × 10^9^ spores/g ^24^ *B. coagulans*) 3 tablets/d	Excipient (no data on used compounds) 3 tablets/d	4 weeks	Significant improvement of IBS symptom
de Chambrun et al. (2015) [108]	The randomized, single-center, double-blind, placebo-controlled, parallel-group clinical study	Rome III	179 males/females 18–75 y	All	8 × 10^9^ cfu/g *S. cerevisiae* CNCM I-3856 1 capsule/d	Dibasic calcium phosphate 1 capsule/d	2 weeks of the run-in period, then 8 weeks of treatment, and 3 weeks of follow-up	Reduced severity of abdominal pain/discomfort The relief of symptoms was limited to the duration of treatment
Yoon et al. (2015) [120]	The randomized, double-blind, placebo-controlled trial	Rome III	81 males/females 19–75 y	All	LacClean Gold-S^®^ (maltodextrin, corn starch, silicon dioxide, *B. bifidum* KCTC 12199BP, *B. lactis* KCTC11904BP, *B. longum* KCTC 12200BP, *Lb. acidophilus* KCTC 11906BP, *Lb. rhamnosus* KCTC 12202BP, *S. thermophilus* KCTC 11870BP; a total of 5 × 10^9^ viable cells/capsule) 2 capsules/d	Maltodextrin, corn starch, silicon dioxide 2 capsules/d	4 weeks	Increased abundance of administrated probiotic strains in fecal samples Amelioration of diarrhea-predominant symptoms of IBS
Majeed et al. (2016) [102]	The randomized, double-blind, parallel-group, placebo-controlled, multi-centered study	Rome III	36 males/females 18–55 y	IBS-D	Microcrystalline cellulose, starch, sodium starch glycolate, magnesium stearate, and *B. coagulans* MTCC 5856 2 × 10^9^ spores/tablet ^25^ 1 tablet/d	Maltodextrin 1 tablet/d	90 days	Attenuation of symptoms (bloating, vomiting, stool frequency, abdominal pain, diarrhea) Improved quality of life
Mezzasalma et al. (2016) [121]	The randomized, double-blind, placebo-controlled study	Rome III	157 males/females 18–65 y	IBS-C	Product 1: 5 × 10^9^ cfu *Lb. acidophilus* DSM 24936, 5 × 10^9^ cfu *Lb. reuteri* DSM 25175, inulin, silica, talc; product 2: 5 × 10^9^ cfu *Lb. rhamnosus* DSM 25568, 5 × 10^9^ cfu *Lb. plantarum* DSM 24937, 5 × 10^9^ cfu *B. lactis* DSM 25566, inulin, silica, talc	Inulin, silica, talc	60 days of treatment, then 30 days of follow-up	Increasing abundance of tested strains in fecal samples during treatment Probiotic strains remained in the stool samples during follow-up period, except *B. lactis* Both products diminish severity of IBS-C symptoms
Spiller et al. (2016) [109]	The multi-center, randomized, double-blind, placebo-controlled trial	Rome III	379 males/females 18–75 y	All	*S. cerevisiae* I-3856 2 capsules/d	No data on used compounds 2 capsules/d	2-week run-in period, then 12 weeks of treatment	Improved GIT symptoms of IBS-C subjects
Compare et al. (2017) [98]	Ex vivo study	Rome III	20 males/females 18–70 y	PI-IBS/IBS-D	*Lb. casei* DG and its postbiotic	Healthy controls (10 out of 20)	na	Decreased inflammatory mucosal response in ex vivo model of PI-IBS-D subjects
Cremon et al. (2018) [87]	The multicenter, randomized, double-blind, cross-over, placebo-controlled, pilot trial	Rome III criteria	40 males/females 18–65 y	All	Gelatin capsule containing 2.4 × 10^10^ viable cells of *Lb. paracasei* CNCM I-1572 2 capsules/d	No data on used compounds 2 capsules/d	2 weeks of the run-in period, next 4 weeks of treatment (probiotic/placebo), followed by 4 weeks of wash-out phase, and another 4 weeks of alternate treatment (placebo/probiotic), then 4-week follow-up	No significant improvement of IBS symptoms compared to placebo Reduced *Ruminococcus* spp. Diminished levels of pro-inflammatory cytokines IL-6, and IL-15 Increased levels of butyrate
Hod et al. (2018) [122]	The randomized, double-blind, placebo-controlled, parallel-group clinical trial	Rome III	97 females 18–70	IBS-D	BIO-25 (3.0 × 10^9^ cfu/capsule *Lb. rhamnosus* LR5; 2.0 × 10^9^ cfu/capsule *Lb. casei* LC5; 1.0 × 10^9^ cfu/capsule *Lb paracasei* LPC5; 1.0 × 10^9^ cfu/capsule *Lb. plantarum* LP3; 5.0 × 10^9^ cfu/capsule *Lb. acidophilus* LA1; 4.0 × 10^9^ cfu/capsule *B. bifidum* BF3; 1.0 × 10^9^ cfu/capsule *B. longum* BG7; 2.0 × 10^9^ cfu/capsule *B. breve* BR3; 1.0 × 10^9^ cfu/capsule *B infantis* BT1; 2.0 × 10^9^ cfu/capsule *S. thermophilus* ST3; 3.0 × 10^9^ cfu/capsule *Lb. bulgaricus* LG1, 3.0 × 10^9^ cfu/capsule *L. lactis* SL6) 2 capsules/d	Cellulose2 capsules/d	2 weeks of run-in period, then 8 weeks of treatment	No effect on fecal microbiota diversity Increased abundance of *Lactobacillus* spp. and *Lactococcus* spp. in stool samples of subjects whose abdominal pain and bloating were reduced Individuals with decreased abdominal pain and improved stool consistency showed a decrease in *Bilophila* genus prevalence
Ishaque et al. (2018) [123]	The randomized, double-blind, placebo-controlled, equal allocation ratio, parallel-group, clinical trial	Rome III	360 males/females 18–55 y	IBS-D	Bio-Kult^®^ (*B. subtilis* PXN 21, *B. bifidum* PXN 23, *B. breve* PXN 25, *B. infantis* PXN 27, *B. longum* PXN 30, *Lb. acidophilus* PXN 35, *Lb. delbrueckii* spp. *bulgaricus* PXN39, *Lb. casei* PXN 37, *Lb. plantarum* PXN 47, *Lb. rhamnosus* PXN 54, *Lb.helveticus* PXN 45, *Lb. salivarius* PXN 57, *L. lactis* PXN 63, *S. thermophilus* PXN 66; a total of 2.0 × 10^9^ cfu/capsule) 4 capsules/d	Microcrystalline cellulose, hydroxypropyl methylcellulose4 capsules/d	16 weeks	Significantly improved IBS symptoms and their severity
Lee et al. (2018) [114]	The single-arm, open-label, pilot study	Rome III	11 males 19–70 y	IBS-D	Ther-Biotic^®^ Complete (*Lb. rhamnosus* 6.0 × 10^9^ cfu/capsule, *B. bifidum* 5.0 × 10^9^ cfu/capsule, *Lb. acidophilus* 3.0 × 10^9^ cfu/capsule, *Lb. casei* 2.5 × 10^9^ cfu/capsule, *Lb. plantarum* 2.0 × 10^9^ cfu/capsule, *Lb. salivarius* 2.0 × 10^9^ cfu/capsule, *B. longum* 1.0 × 10^9^ cfu/capsule, *S. thermophilus* 1.0 × 10^9^ cfu/capsule, *Lb. bulgaricus* 1.0 × 10^9^ cfu/capsule, *Lb. paracasei* 5.0 × 10^8^ cfu/capsule, *B. lactis* 5.0 × 10^8^ cfu/capsule, *B. breve* 5.0 × 10^8^ cfu/capsule)	No placebo or control group	8 weeks	Ameliorated abdominal discomfort, dyspepsia, flatulence, and stool consistency Decreased SIBO prevalence Beneficial impact on GIT microbiota
Kim et al. (2018) [96]	The double-blind, randomized, placebo-controlled, parallel study	individual protocol	42 males/females 20–54 y	wd	*Lb. gasseri* BNR17 Low dose: 2 × 10^8^ cfu/d ^26^ Medium dose: 2 × 10^9^ cfu/d High dose: 2 × 5 × 10^9^ cfu/d	Dextrin	7 days of the run-in period, then 4 weeks	Amelioration of IBS symptoms The abundance of probiotic strain in fecal samples of treated subjects The optimal dosage appeared to be high dose (2 × 5 × 10^9^ cfu/d)
Majeed et al. (2018) [127]	The randomized, double-blind, placebo-controlled, multicenter, parallel-group clinical study	Rome III	40 males/females 20–65 y	wd	Microcrystalline cellulose, starch, sodium starch glycolate, magnesium stearate, 2.0 × 10^9^ spores/tablet *B. coagulans* MTCC 5856 1 tablet/d	Microcrystalline cellulose, starch, sodium starch glycolate, magnesium stearate1 tablet/d	90 days of treatment, then 15 days of follow-up	Decrease in depression, along with diminished IBS symptoms Reduced sleeplessness Decreased serum levels of myeloperoxidase
Shin et al. (2018) [97]	The single-center, randomized, double-blind, placebo-controlled clinical trial	Rome III	51 males/females 20–55 y	IBS-D	Maltodextrin, microcrystalline cellulose, magnesium stearate, *Lb. gasseri* BNR17 (total of 10^10^ cfu/d) 4 capsules/d	Maltodextrin, microcrystalline cellulose, magnesium stearate4 capsules/d	8 weeks	Improved bowel habits (longer colon transit time) Positive impact on intestinal microbiota (decreased *Firmicutes*, increased *Actinobacteria*, and *Bacteroidetes*)
Sun et al. (2018) [105]	The prospective, multicenter, randomized, double-blind, placebo-controlled trial	Rome III criteria	200 males/females 18–65 y	IBS-D	1.5 × 10^7^ cfu/capsule *C. butyricum* 9 capsules/d	No data on used compounds 9 capsules/d	4 weeks	Improvement in overall IBS symptoms and quality of patients’ life No significant changes in intestinal microbiota diversity Reduction of *Clostridium sensu stricto* in microbial community Improvement of alanine and tryptophan metabolism
Hong et al. (2019) [111]	Animal study	na	54 males C57L/B6 mice (15.5 ± 1.0 g) aged 4 weeks	na PI-IBS model (*Trichinella*)	Formulated probiotics (DW; *Lb. acidophilus* LA5, *B. lactis* BB12, *S. boulardii*) 0.2 mL of the solution of 5.0 × 10^9^ cfu/g VSL#3 0.2 mL of the solution of 5.0 × 10^9^ cfu/g	No data on used compounds 0.2 mL of the solution of 5.0 × 109 cfu/g	4 weeks	Decreased levels of serum pro-inflammatory cytokines in uninfected and infected mice treated with probiotics (DW and VSL#3) Diminished visceral hypersensitivity (DW)
Leventogiannis et al. (2019) [112]	Open-label clinical study	Rome III/SIBO-positive or SIBO-negative	Males/Females ≥ 18 y	All	Lactolevure^®^ (1.5 × 10^9^ cfu/capule *S. boulardii*, 1.75 × 10^9^ cfu/capsule *B. lactis* BB-12, 1.5 × 10^9^ cfu/capsule *Lb. acidophilus* LA-5, 5.0 × 10^8^ cfu/capsule *Lb. plantarum*) 2 capsules/d	No placebo/control group	30 days	Amelioration of bloating More significant improvement of symptoms among IBS patients with SIBO Decreased abdominal pain severity
Madempudi et al. (2019) [103]	The randomized, double-blind, placebo-controlled trial	Rome III	108 males/females 18–60 y	wd	Excipient (maltodextrin), 2.0 × 10^9^ cfu/capsule B. coagulans Unique IS2	Excipient (maltodextrin)	8 weeks	Relief in the severity of symptoms (bloating, incomplete evacuation, urgency, straining, the passage of gas, bowel habit satisfaction, and stool consistency) Reduced abdominal pain Increased number of complete spontaneous bowel movement
Oh et al. (2019) [124]	The randomized, double-blind, placebo-controlled trial	Rome III	50 males/females 19–60 y	With the exclusion of IBS-C	Foodis Lactobacillus—excipients (olive oil, pine oil), and *Lb. paracasei*, *Lb. salivarius*, 1 × 10^9^ cfu/mL *Lb. plantarum* (ratio 5:4:1)	Excipients (olive oil, pine oil)	1 week screening period, then 4 weeks of treatment	Relief in global IBS symptoms Decreased severity of abdominal pain
Zhang et al. (2019) [125]	Pilot study	Rome III	15 males/females 18–65 y	IBS-D	Bifico^®^ (*B. longum*, *Lb. acidophilus*, *E. faecalis;* a total of ≥1.0 × 10^7^ cfu)20 mg 3 times a day	Antidepressant (Duloxetine) 30 mg/d for 4 days, then 60 mg/d	8 weeks	Changes in gut microbiota and SCFAs ^27^ concentrations (probiotic and antidepressant) Reduced severity of abdominal symptoms (probiotic and antidepressant) Decreased plasma levels of cytokines (probiotic and antidepressant)
Zhao et al. (2019) [106]	Animal studies	na	24 C57BL/6 male mice aged 6–8 weeks	na PI-IBS model (TNBS ^28^)	1 × 10^8^ cfu/mL *C. butyricum* 200 µL/d	Saline	4 weeks of model preparation, one week of treatment	Attenuated intestinal visceral hypersensitivity Diminished low-grade mucosal inflammation (suppressed production of cytokines, decreased number of lamina propria dendritic cells)
Caviglia et al. (2020) [93]	The prospective study	Rome IV	16 males/females 16–65 y	IBS-D	*B. longum* ES1 1 × 10^9^ cfu	Lack of control	8–12 weeks	Improvement in general IBS symptoms Amelioration of the immune-inflammatory condition (reduced cytokines, and zonulin levels) Increased integrity of the intestinal barrier
Lewis et al. (2020) [95]	The randomized, double-blind, placebo-controlled, 3-arm parallel-group study	Rome III	285 males/females ≥ 18 y	All	Excipients (potato starch, and magnesium stearate), and 10 × 10^9^ cfu/capsule *of B. longum* HA-196, or *Lb. paracasei* R0175	Potato starch, and magnesium stearate	2-week run-in period, next 8 weeks of treatment	Significantly improved bowel habits, and stool consistency in IBS-D, and IBS-C subjects (*Lb. paracasei* R0175) The positive impact of social aspects of life (both probiotic strains) Increased number of *Bifidobacterium* ssp. in fecal samples (*B. langum* HA-196)No vital changes in *A. muciniphila*, and *F. prausnitzii* abundance Distinct placebo effect
Martoni et al. (2020) [92]	The randomized, double-blind, placebo-controlled, multicenter study	Rome IV	336 males/females 18–70 y	wd	Microcrystalline cellulose, and ≥1 × 10^10^ cfu/capsule of *Lb. acidophilus* DDS^®^-1, or *B. animalis* subsp. *lactis* UABla-12™ 1 capsule/d	Microcrystalline cellulose 1 capsule/d	2-week run-in period (only placebo), 6 weeks of treatment (probiotic/placebo)	Improvement in stool consistency Reduced abdominal pain severity Vital amelioration of IBS symptoms Diminished stress levels (*Lb. acidophilus* DDS^®^-1)
Zhou et al. (2020) [94]	Animal study	na	Male Sprague-Dawley rats (weight: 225~260 g)	na WAS model	*B. longum* 1 × 10^9^ cfu/mL once a day	0.9% saline	10 days	Influenced Paneth cells function—enhanced lysozyme production, and repair of mucus No difference in serum cytokines levels Beneficial alteration of GIT microbiota Improved intestinal permeability
Gupta et al. (2021) [104]	The prospective, interventional, randomized, double-blind, placebo-controlled clinical study	Rome IV	40 males/females 18–65 y	wd	Excipient with *B. coagulans* LBSC 2 × 10^9^ spores/sachet 3 sachets/d ^29^	Excipient with maltodextrin 3 sachets/d	Up to 80 days	Improvement in abdominal symptoms (pain, stomach rumbling) Attenuation of bloating, cramping, nausea, vomiting, diarrhea, anxiety Beneficial modulation of GIT microbiota Amelioration in stool consistency
Seong et al. (2021) [99]	Animal study	na	14 male Wistar rats (weight: 304 ± 1.4 g) aged 8 weeks	na IBS induced by chronic restraint stress	Maltodextrin and heat-killed 1 × 10^11^ cfu *Lb. casei* DKGF7	Maltodextrin	4 weeks	Improvement of IBS symptoms in the animal model Decrease in serum corticosterone levels, inflammatory cytokines in colonic tissue Enhanced expression of tight junction proteins
Skrzydło-Radomańska et al. (2021) [126]	The randomized, double-blind, placebo-controlled, parallel-group trial	Rome III	51 males/females 8–75 y	IBS-D	NordBiotic™ (*B. breve* BB010 *B. longum* BL020, *B. bifidum* BF030, *B. lactis* BL040, *Lb. rhamnosus* LR110, *Lb paracasei* LPC100, *Lb acidophilus* LA120, *Lb. casei* LC130, *Lb plantarum* LP140, *S. thermophilus* ST250; Total 2.5 × 10^9^ cfu/capsule) 2 capsules/d	Maltodextrin 2 capsules/d	8 weeks	Improved quality of patients’ life Diminished severity of abdominal pain

^1^ the final (after screening) number of participants who received treatment is given; ^2^ years old; ^3^ without differentiation; ^4^ colony-forming units per milliliter, ^5^ milliliters per day; ^6^ capsules per day; ^7^ not applicable; ^8^ small intestinal bacterial overgrowth; ^9^ early rise in breath hydrogen after lactulose; ^10^ colony-forming units per packet; ^11^ packets per day; ^12^ colony-forming unit per capsule; ^13^ colony-forming units per gram; ^14^ cells per day; ^15^ milligrams per day; ^16^ post-infection IBS; ^17^ post-antibiotic IBS; ^18^ tablets per day; ^19^ colony-forming units per cup; ^20^ cups per day; ^21^ interleukins; ^22^ tumor necrosis factor α; ^23^ milliliters per kilogram; ^24^ spores per gram; ^25^ spores per tablet; ^26^ colony-forming units per day; ^27^ short-chain fatty acids; ^28^ 2,4,6-trinitrobenzenesulfonic acid; ^29^ sachets per day.

**Table 2:** Studies on the effect of prebiotics on IBS individuals.

Paper	Research Type	Participants Selection Criteria	Participants ^1^	IBS Subtype	Preparation (Dosage)	Placebo/Control (Dosage)	Duration	Outcomes
Hunter et al. (1999) [52]	The randomized, controlled, double-blind, crossover study	Individual protocol	21 males/females 18–65 y ^2^	wd ^3^	Oligofructose (Raftilose P95) three times 2 g/d ^4^	Sucrose three times 1 g/d	First 2 weeks of normal diet, then 2 weeks of controlled standard UK diet (45% carbohydrate, 40% fat, and 15% protein)	No significant results
Olesen et al. (2000) [128]	The prospective, randomized, placebo-controlled, single-blind, and double-blind phases	Manning	98 males/females 18–70 y	wd	FOS ^5^ 10 g/d (2 weeks)/20 g/d (next 10 weeks)	Glucose 10 g (2 weeks)/20 g/d (next 10 weeks)	First 2 weeks of single-blind phase (only placebo), then 12 weeks of double-blind phase (prebiotic/placebo)	FOS may increase the severity of IBS symptoms; however, patients might adapt after prolonged usage
Silk et al. (2009) [130]	The single-center, parallel, patient blinded, randomized, crossover, placebo-controlled trial	Rome II	44 males/females 20–79 y	All	*trans*-GOS ^6^ 3.5 or 7.0 g/d	Maltodextrins DE 20 3.5 or 7.0 g/d	Baseline period—2 weeks (only placebo); next phase—3 months (prebiotic/placebo)	Increased number of *Bifidobacterium* spp. to a level comparable with healthy people (both doses of GOS) Increased number of *Eubacterium rectale/C.coccoides* (GOS dose of 3.5 g/d) Reduced number of *C. perfringens* (GOS dose of 7.0 g) Changes in the consistency of feces, flatulence, and overall improvement of IBS symptoms (GOS) The reduced anxiety level in the IBS-D group (GOS dose of 7.0 g/d)
Azpiroz et al. (2016) [129]	The parallel, placebo-controlled, randomized, double-blind study	Rome III	79 males/females 18–60 y	All	scFOS two times 2.5 g/d	Maltodextrins two times 2.5 g/d	28 days	Increased number of *Bifidobacterium* spp. Decreased level of anxiety Attenuated severity of IBS symptoms No effect on rectal hypersensitivity
Niv et al. (2016) [132]	The prospective, randomized, double-blind, placebo-controlled study	Rome III	108 males/females 18–77 y	All	PHGG 3 g/d (first week)/6 g/d (11 weeks)	Maltodextrin 3 g/d (first week)/6 g/d (11 weeks)	First 2 weeks without prebiotic/placebo, next 12 weeks of product administration, then 4 weeks of follow-up	Improvement on bloating and gasses
Chen et al. (2017) [131]	Animal study	na ^7^	60 four-week-old female specific pathogen-free (SPF) C57BL/6 mice	na PI-IBS ^8^ model	The PB (FOS, GOS, inulin, and anthocyanins) 1.26 mg/g body weight	Saline/healthy control group	8 weeks of preventive administration of the PB/saline before infection with *Trichinella spiralis* larvae, then 8 weeks for recovery without prebiotic	Faster recovery from body weight loss Pretreatment with PB could help to improve the well-being of PI-IBS patients PB can diminish inflammation both in the Caco-2 cells and the IBS mice model Protection of intestinal barrier integrity PB could help protect the homeostasis of GIT microbiota

^1^ the final (after screening) number of participants who received treatment is given; ^2^ years old; ^3^ without differentiation; ^4^ gram per day; ^5^ fructooligosaccharides; ^6^ galactooligosaccharides; ^7^ not applicable; ^8^ post-infection IBS.

**Table 3:** Researches on the influence of synbiotics on IBS patients.

Paper	Research Type	Participants Selection Criteria	Participants ^1^	IBS Subtype	Preparation (Dosage)	Placebo/Control (Dosage)	Duration	Outcomes
Min et al. (2012) [141]	The randomized, double-blind, controlled trial	Rome III	117 males/females 18–70 y ^2^	All	Yogurt with standard strains *S. thermophilus* (≥3 × 10^9^ cfu ^3^/bottle) and *Lb. acidophilus* (≥10^9^ cfu/bottle) with the addition of *Bifidobacterium animalis* subsp. *lactis* Bb-12 (≥10^11^ cfu/bottle), *Bifidobacterium* enhancer, and acacia dietary fiber	Yogurt with standard strains *S. thermophilus* (≥3 × 10^9^ cfu/bottle) and *Lb. acidophilus* (≥10^9^ cfu/bottle) with the addition of *Bifidobacterium animalis* subsp. *lactis* Bb-12 (≥10^11^ cfu/bottle)	8 weeks	Improvement in bowel habits and IBS symptoms
Cappello et al. (2013) [134]	The parallel-group, double-blinded, placebo-controlled study	Rome III	64 males/females 18–75 y	wd ^4^	Probinul ^®^ (5 × 10^9^ *Lb. plantarum*, 2 × 10^9^ *Lb. casei* subp. *rhamnosus*, 2 × 10^9^ *Lb. gasseri*, 1 × 10^9^ *B. infantis*, 1 × 10^9^ *B. longum*, 1 × 10^9^ *Lb. acidophilus*, 1 × 10^9^ *Lb. salivarus*, 1 × 10^9^ *Lb. sporogenes*, 5 × 10^9^ *S. thermophilus*, 2 g inulin, 1.3 g tapioca-resistant starch) twice 5 g/d ^5^	No data on used compounds product by CaDi Group (Rome, Italy)	2 weeks prior to synbiotic/placebo administration, then 4 weeks of treatment	Decreased flatulenceThe increased transition time of intestinal content Improved in self-scored quality of IBS patients’ life No overall relief of symptoms
Bucci et al. (2014) [137]	The parallel-group, double-blinded, randomized, placebo-controlled study (core study) and the open-label prospective, partially controlled	Rome III	64 males/females 18–75 y	wd	Probinul^®^ twice 5 g/d (during extension period only 2 weeks/month)	Lack of information	4 weeks of core study, then 6 months of the extension period	Decreased flatulence even during cyclic administration
Rogha et al. (2014) [54]	The randomized, double-blinded, placebo-controlled trial	Rome III	56 males/females The average age of 39.8 ± 12.7	wd	Lactol^®^ (1.5 × 10^8^ spores of *B. coagulans* and FOS) 100 mg	Lactose starch and tartazine 100 mg	12 weeks	Relief from abdominal pain/discomfort and diarrhea
Shavakhi et al. (2014) [135]	The randomized, placebo-controlled, triple-blinded study	Rome II	129 males/females average age of 36.2 ± 9.3	wd	Balance^®^ (FOS, magnesium stearate, hydroxypropyl methyl cellulose, *Lb. casei*, *Lb. rhamnosus*, *Lb. acidophilus*, *Lb. bulgaricus*, *B. breve*, *B. longum*, *S. thermophilus*; a total of 1 × 10^8^ cfu/capsule) 2 capsules a day	No data on used compounds 2 capsules a day	14 days	No effect of treatment
Bogovič Matijašic et al. (2016) [136]	The double-blind, randomized, placebo-controlled multicenter trial	Rome III	30 subjects 18–65 y	IBS-C	Fermented milk (starter culture: *S. thermophilus* ABT-21, probiotics: 1.8 × 10^7^ cfu/g Lb acidophilus La-5, 2.5 × 10^7^ cfu/g B. lactis BB-12, 2% dietary fiber Beneo Orafti Synergy1—90% inulin, 10% oligofructose) 360 g/d	Heat-treated fermented milk without probiotic bacteria and dietary fibers 360 g/d	2 weeks run-in period, then 4 weeks of treatment, and 2 weeks of follow-up	Increased abundance of used probiotic strains in subjects’ fecal samples Transient colonization of used probiotics The abundance of the *Enterobacteriaceae* family was not affected No significant changes in fecal microbiota
Moser et al. (2019) [139]	Pilot study	Individual protocol	10 males/females 37–53 y	IBS-D	OMNi-BiOTiC^®^ Stress Repair (corn starch, maltodextrin, inulin, FOS, potassium chloride, magnesium sulfate, mangan sulfate, enzymes, 7.5 × 10^9^ of each strain: *Lb. casei* W56, *Lb. acidophilus* W22, *Lb. paracasei* W20, *Lb. salivarius* W24, *Lb. plantarum* W62, *L. lactis* W19, *B. lactis* W51, *B. lactis* W52, *B. bifidum* W23)	na ^6^	4 weeks	Increased phylogenetic diversity of gastric and duodenal microbiota Reduced number of CD4+ T cells in the ascending colon Higher levels of acetate and butyrate in fecal samples
Lee et al. (2019) [140]	The single-center, randomized, double-blind, placebo-controlled clinical trial	Rome III	28 males/females ≥19 y	All	Ultra-Probiotics-500 (1 × 10^13^ cfu of probiotic strains: *Lactobacillus* (*rhamnosus*, *acidophilus*, *casei*, *bulgaricus*, *plantarum*, and *salivarius*), *Bifidobacterium* (*bifidum* and *longum*, 175 mg of FOS, 150 mg of *Ulmus davidiana*, 10 mg of *Geum urbanum*, and 100 mg of inulin) 1 capsule/d (low-dose group)2 capsules/d (high-dose group)	The same material used for encapsulation. No data on used compounds. 1 capsule/d (low-dose group) 2 capsules/d (placebo group)	8 weeks	No dose-dependent effects Decrease the fatigue in IBS individuals (high-dose) Relief in abdominal pain/discomfort, bloating, stool patterns
Bahrudin et al. (2020) [142]	The prospective, double-blind, randomized, controlled trial	Rome III	163 males/females 22–37 y	IBS-C	Synbiotic drink (water, sugar, skimmed milk powder (cow), stabilizers (polydextrose), fermented milk (water, acidity regulator, skimmed milk powder (cow), and lactobacillus), acidity regulators, soybean fiber, and flavoring) with *Lb. helveticus* and 1.5 g/100 mL polydextrose 350 mL/d ^5^	Probiotic drink ((water, sugar, skimmed milk powder (cow), stabilizers (polydextrose), fermented milk (water, acidity regulator, skimmed milk powder (cow), and lactobacillus), acidity regulators, soybean fiber, and flavoring)) with *Lb. helveticus* 350 mL/d	1 week	Shortened intestinal transition time Reduced fecal pH Relief in constipation-related symptoms No difference between synbiotic and control group—probiotic alone conferred a health benefit
Seong et al. (2020) [144]	Animal study	na	20 male Wistar rats (weight: 350 ± 50 g) aged 8 weeks	na	Treatment group 1—maltodextrin, 1 × 10^10^ cfu/g *Lb. paracasei* DKGF; Treatment group 2—maltodextrin, 1 × 10^10^ cfu/g *Lb. paracasei* DKGF, 10.0 mg (*w*/*w*) *Opuntia* extract; Treatment group 3—maltodextrin, 1 × 10^10^ cfu/g *Lb. paracasei* DKGF, 30.0 mg (*w*/*w*) *Opuntia* extract	Maltodextrin	4 weeks	Improved stool consistency (better in synbiotic groups) Decreased serum corticosterone levels (lower in synbiotic groups) Low levels of TNF-α ^7^ in the colonic mucosa (both synbiotic and probiotic groups) Increased expression of the tight junction proteins (higher in synbiotic groups) Higher abundance of *Lb. paracasei* in fecal samples (more significant difference in synbiotic groups)
Skrzydło-Radomańska et al. (2021) [143]	The randomized, double-blind, placebo-controlled, parallel group trial	Rome III	68 males/females 18–60 y	IBS-D	Synbiotic containing a total of 5.0 × 10^9^ probiotic strains (*B. lactis* DSMZ 32269, *B. longum* DSMZ 32946, *B. bifidum* DSMZ 32403, *Lb. rhamnosus* FloraActive 19070-2, *Lb. acidophilus* DSMZ 32418) and 947 mg of scFOS 2 sachet/d	978 mg of maltodextrin2 sachet/d	2 weeks screening period, then 8 weeks of treatment	Attenuation of IBS symptoms (pain, flatulence, stool pressure, feeling of incomplete bowel movements) Decreased severity of IBS symptoms

^1^ the final (after screening) number of participants who received treatment is given; ^2^ years old; ^3^ colony-forming units; ^4^ without differentiation; ^5^ not applicable; ^6^ milliliters per day; ^7^ tumor necrosis factor α.

## References

[1] Ford A.C., Sperber A.D., Corsetti M., Camilleri M. (2020). Irritable Bowel Syndrome. Lancet.

[2] Üçüncü M.Z., Çoruh Akyol B., Toprak D. (2020). The Early Diagnosis of Fibromyalgia in Irritable Bowel Syndrome Patients. Med. Hypotheses.

[3] Hadjivasilis A., Tsioutis C., Michalinos A., Ntourakis D., Christodoulou D.K., Agouridis A.P. (2019). New Insights into Irritable Bowel Syndrome: From Pathophysiology to Treatment. Ann. Gastroenterol..

[4] Mosińska P., Sałaga M., Fichna J. (2017). Clinical Features of Irritable Bowel Syndrome. Introduction to Gastrointestinal Diseases.

[5] Ida M., Nishida A., Akiho H., Nakashima Y., Matsueda K., Fukudo S. (2017). Evaluation of the Irritable Bowel Syndrome Severity Index in Japanese Male Patients with Irritable Bowel Syndrome with Diarrhea. BioPsychoSoc. Med..

[6] Mokha J.S., Hyams J.S., Faure C., Thapar N., di Lorenzo C. (2017). Irritable Bowel Syndrome. Pediatric Neurogastroenterology Gastrointestinal Motility and Functional Disorders in Children.

[7] Israel E.J., Kaplan J.L., Fiechtner L., Goldstein M.A. (2017). Gastroenterology and Nutrition: Healthy Eating in Adolescence and Nutritional Supplements; Irritable Bowel Syndrome; Inflammatory Bowel Disease. The MassGeneral Hospital for Children Adolescent Medicine Handbook.

[8] Van Malderen K., De Winter B.Y., De Man J.G., De Schepper H.U., Lamote K. (2020). Volatomics in Inflammatory Bowel Disease and Irritable Bowel Syndrome. EBioMedicine.

[9] Rutsch A., Kantsjö J.B., Ronchi F. (2020). The Gut-Brain Axis: How Microbiota and Host Inflammasome Influence Brain Physiology and Pathology. Front. Immunol..

[10] Pazhouh H.K., Hosseini S.M., Taghipour A., Hamedi S., Noras M. (2020). Anti-Irritable Bowel Syndrome Syrup Improves Constipation-Predominant Irritable Bowel Syndrome: A Randomized, Placebo-Controlled Trial. Chin. J. Integr. Med..

[11] Staudacher H.M., Mikocka-Walus A., Ford A.C. (2021). Common Mental Disorders in Irritable Bowel Syndrome: Pathophysiology, Management, and Considerations for Future Randomised Controlled Trials. Lancet Gastroenterol. Hepatol..

[12] Shah K., Ramos-Garcia M., Bhavsar J., Lehrer P. (2020). Mind-Body Treatments of Irritable Bowel Syndrome Symptoms: An Updated Meta-Analysis. Behav. Res. Ther..

[13] Su X.-T., Wang L.-Q., Zhang N., Li J.-L., Qi L.-Y., Wang Y., Yang J.-W., Shi G.-X., Liu C.-Z. (2021). Standardizing and Optimizing Acupuncture Treatment for Irritable Bowel Syndrome: A Delphi Expert Consensus Study. Integr. Med. Res..

[14] Tang Z.P. (2009). Traditional Chinese Medicine Clinical Experience of the Treatment for Irritable Bowel Syndrome. Chin. J. Integr. Med..

[15] Herndon C.C., Wang Y.P., Lu C.L. (2020). Targeting the Gut Microbiota for the Treatment of Irritable Bowel Syndrome. Kaohsiung J. Med. Sci..

[16] FAO, WHO (2002). Guidelines for the Evaluation of Probiotics in Food.

[17] Hill C., Guarner F., Reid G., Gibson G.R., Merenstein D.J., Pot B., Morelli L., Canani R.B., Flint H.J., Salminen S. (2014). Expert Consensus Document: The International Scientific Association for Probiotics and Prebiotics Consensus Statement on the Scope and Appropriate Use of the Term Probiotic. Nat. Rev. Gastroenterol. Hepatol..

[18] Gibson G.R., Hutkins R., Sanders M.E., Prescott S.L., Reimer R.A., Salminen S.J., Scott K., Stanton C., Swanson K.S., Cani P.D. (2017). Expert Consensus Document: The International Scientific Association for Probiotics and Prebiotics (ISAPP) Consensus Statement on the Definition and Scope of Prebiotics. Nat. Rev. Gastroenterol. Hepatol..

[19] Núñez-Sánchez M.A., Herisson F.M., Cluzel G.L., Caplice N.M. (2021). Metabolic Syndrome and Synbiotic Targeting of the Gut Microbiome. Curr. Opin. Food Sci..

[20] Schofield P.F., Haboubi N.Y., Martin D.F., Schofield P.F., Haboubi N.Y., Martin D.F. (1993). Irritable Bowel Syndrome. Highlights in Coloproctology.

[21] Ritchie J.A. (1970). The Transport of Colonic Contents in the Irritable Colon Syndrome. Gut.

[22] Chaudhary N.A., Truelove S.C. (1962). The Irritable Colon Syndrome: A Study of the Clinical Features, Predisposing Causes, and Prognosis in 130 Cases. QJM Int. J. Med..

[23] Hawkins H.P. (1906). The Reality of Enterospasm and Its Mimicry of Appendicitis. Br. Med. J..

[24] Ryle J.A. (1928). Chronic Spasmodic Affections of The Colon and The Disease Which They Simulate. Lancet.

[25] Scarlett E.P. (1937). Functional Disturbances of the Colon: The Irritable (Spastic) Colon. Can. Med. Assoc. J..

[26] Gallagher D.M. (1961). Evaluation of Colon Dysfunction. Calif. Med..

[27] Misiewicz J.J., Waller S.L., Eisner M. (1966). Motor Responses of Human Gastrointestinal Tract to 5-Hydroxytryptamine in Vivo and in Vitro. Gut.

[28] Madden J.A.J., Hunter J.O. (2002). A Review of the Role of the Gut Microflora in Irritable Bowel Syndrome and the Effects of Probiotics. Br. J. Nutr..

[29] Neal K.R., Hebden J., Spiller R. (1997). Prevalence of Gastrointestinal Symptoms Six Months after Bacterial Gastroenteritis and Risk Factors for Development of the Irritable Bowel Syndrome: Postal Survey of Patients. Br. Med. J..

[30] García Rodríguez L.A., Ruigómez A. (1999). Increased Risk of Irritable Bowel Syndrome after Bacterial Gastroenteritis: Cohort Study. Br. Med. J..

[31] Malinen E., Rinttilä T., Kajander K., Mättö J., Kassinen A., Krogius L., Saarela M., Korpela R., Palva A. (2005). Analysis of the Fecal Microbiota of Irritable Bowel Syndrome Patients and Healthy Controls with Real-Time PCR. Am. J. Gastroenterol..

[32] Kassinen A., Krogius-Kurikka L., Mäkivuokko H., Rinttilä T., Paulin L., Corander J., Malinen E., Apajalahti J., Palva A. (2007). The Fecal Microbiota of Irritable Bowel Syndrome Patients Differs Significantly from That of Healthy Subjects. Gastroenterology.

[33] Connell A.M. (1968). The Irritable Colon Syndrome. Postgrad. Med. J..

[34] Manousos O.N., Truelove S.C., Lumsden K. (1967). Transit Times of Food in Patients with Diverticulosis or Irritable Colon Syndrome and Normal Subjects. Br. Med. J..

[35] Cann P.A., Read N.W., Brown C., Hobson N., Holdsworth C.D. (1983). Irritable Bowel Syndrome: Relationship of Disorders in the Transit of a Single Solid Meal to Symptom Patterns. Gut.

[36] Manning A.P., Heaton K.W., Thompson W.G., Morris A.F. (1978). Towards Positive Diagnosis of the Irritable Bowel. Br. Med. J..

[37] Lacy B., Patel N. (2017). Rome Criteria and a Diagnostic Approach to Irritable Bowel Syndrome. J. Clin. Med..

[38] Thompson W.G. (2006). The Road to Rome. Gastroenterology.

[39] Dotevall G., Groll E. (1974). Controlled Clinical Trial of Mepiprazole in Irritable Bowel Syndrome. Br. Med. J..

[40] MacDougall I.P. (1966). Irritable Colon. J. R. Soc. Med..

[41] Ritchie J.A., Truelove S.C. (1979). Treatment of Irritable Bowel Syndrome with Lorazepam, Hyoscine Butylbromide, and Ispaghula Husk. Br. Med. J..

[42] Diamant N.E. (1977). Diarrhea in the Irritable Colon Syndrome. Can. Med. Assoc. J..

[43] Barber T.M., Kabisch S., Pfeiffer A.F.H., Weickert M.O. (2020). The Health Benefits of Dietary Fibre. Nutrients.

[44] Hall M.J., Barry R.E. (1991). Current Views on the Aetiology and Management of the Irritable Bowel Syndrome. Postgrad. Med. J..

[45] Francis C.Y., Whorwell P.J. (1997). The Irritable Bowel Syndrome. Postgrad. Med. J..

[46] Madia V.N., Messore A., Saccoliti F., Tudino V., De Leo A., De Vita D., Bortolami M., Scipione L., Pindinello I., Costi R. (2020). Tegaserod for the Treatment of Irritable Bowel Syndrome. Anti Inflamm. Anti Allergy Agents Med. Chem..

[47] Crowell M.D. (2004). Role of Serotonin in the Pathophysiology of the Irritable Bowel Syndrome. Br. J. Pharmacol..

[48] Gunn M.C., Cavin A.A., Mansfield J.C. (2003). Management of Irritable Bowel Syndrome. Postgrad. Med. J..

[49] Zheng Y., Yu T., Tang Y., Xiong W., Shen X., Jiang L., Lin L. (2017). Efficacy and Safety of 5-Hydroxytryptamine 3 Receptor Antagonists in Irritable Bowel Syndrome: A Systematic Review and Metaanalysis of Randomized Controlled Trials. PLoS ONE.

[50] Fukudo S., Nakamura M., Hamatani T., Kazumori K., Miwa H. (2021). Efficacy and Safety of 5-HT4 Receptor Agonist Minesapride for Irritable Bowel Syndrome with Constipation in a Randomized Controlled Trial. Clin. Gastroenterol. Hepatol..

[51] Rees W.D.W., Evans B.K., Rhodes J. (1979). Treating Irritable Bowel Syndrome with Peppermint Oil. Br. Med. J..

[52] Hunter J.O., Tuffnell Q., Lee A.J. (1999). Controlled Trial of Oligofructose in the Management of Irritable Bowel Syndrome. J. Nutr..

[53] Ghoshal U.C., Shukla R., Ghoshal U., Gwee K.A., Ng S.C., Quigley E.M.M. (2012). The Gut Microbiota and Irritable Bowel Syndrome: Friend or Foe?. Int. J. Inflamm..

[54] Rogha M., Esfahani M.Z., Zargarzadeh A.H. (2014). The Efficacy of a Synbiotic Containing *Bacillus coagulans* in Treatment of Irritable Bowel Syndrome: A Randomized Placebo-Controlled Trial. Gastroenterol. Hepatol. Bed Bench.

[55] Al Bander Z., Nitert M.D., Mousa A., Naderpoor N. (2020). The Gut Microbiota and Inflammation: An Overview. Int. J. Environ. Res. Public Health.

[56] Matsumoto N., Park J., Tomizawa R., Kawashima H., Hosomi K., Mizuguchi K., Honda C., Ozaki R., Iwatani Y., Watanabe M. (2021). Relationship between Nutrient Intake and Human Gut Microbiota in Monozygotic Twins. Medicina.

[57] Mari A., Abu Baker F., Mahamid M., Sbeit W., Khoury T. (2020). The Evolving Role of Gut Microbiota in the Management of Irritable Bowel Syndrome: An Overview of the Current Knowledge. J. Clin. Med..

[58] Zhuang X., Xiong L., Li L., Li M., Chen M. (2017). Alterations of Gut Microbiota in Patients with Irritable Bowel Syndrome: A Systematic Review and Meta-Analysis. J. Gastroenterol. Hepatol. (Aust.).

[59] Ooi S.L., Correa D., Pak S.C. (2019). Probiotics, Prebiotics, and Low FODMAP Diet for Irritable Bowel Syndrome—What Is the Current Evidence?. Complementary Ther. Med..

[60] Pittayanon R., Lau J.T., Yuan Y., Leontiadis G.I., Tse F., Surette M., Moayyedi P. (2019). Gut Microbiota in Patients With Irritable Bowel Syndrome—A Systematic Review. Gastroenterology.

[61] Chong P.P., Chin V.K., Looi C.Y., Wong W.F., Madhavan P., Yong V.C. (2019). The Microbiome and Irritable Bowel Syndrome—A Review on the Pathophysiology, Current Research and Future Therapy. Front. Microbiol..

[62] Mei L., Zhou J., Su Y., Mao K., Wu J., Zhu C., He L., Cui Y. (2021). Gut Microbiota Composition and Functional Prediction in Diarrhea-Predominant Irritable Bowel Syndrome. BMC Gastroenterol..

[63] Tap J., Derrien M., Törnblom H., Brazeilles R., Cools-Portier S., Doré J., Störsrud S., le Nevé B., Öhman L., Simrén M. (2017). Identification of an Intestinal Microbiota Signature Associated with Severity of Irritable Bowel Syndrome. Gastroenterology.

[64] Su T., Liu R., Lee A., Long Y., Du L., Lai S., Chen X., Wang L., Si J., Owyang C. (2018). Altered Intestinal Microbiota with Increased Abundance of *Prevotella* Is Associated with High Risk of Diarrhea-Predominant Irritable Bowel Syndrome. Gastroenterol. Res. Pract..

[65] Barandouzi Z.A., Lee J., Maas K., Starkweather A.R., Cong X.S. (2021). Altered Gut Microbiota in Irritable Bowel Syndrome and Its Association with Food Components. J. Pers. Med..

[66] Shukla R., Ghoshal U., Dhole T.N., Ghoshal U.C. (2015). Fecal Microbiota in Patients with Irritable Bowel Syndrome Compared with Healthy Controls Using Real-Time Polymerase Chain Reaction: An Evidence of Dysbiosis. Dig. Dis. Sci..

[67] König J., Brummer R.J. (2014). Modulation of the gut ecosystem in irritable bowel syndrome. Pharma Nutrition: An Overview (AAPS Advances in the Pharmaceutical Sciences Series).

[68] Lee S.M., Kim N., Yoon H., Kim Y.S., Choi S.I., Park J.H., Lee D.H. (2021). Compositional and Functional Changes in the Gut Microbiota in Irritable Bowel Syndrome Patients. Gut Liver.

[69] Ahluwalia B., Iribarren C., Magnusson M.K., Sundin J., Clevers E., Savolainen O., Ross A.B., Törnblom H., Simrén M., Öhman L. (2021). A Distinct Faecal Microbiota and Metabolite Profile Linked to Bowel Habits in Patients with Irritable Bowel Syndrome. Cells.

[70] Dlugosz A., Winckler B., Lundin E., Zakikhany K., Sandström G., Ye W., Engstrand L., Lindberg G. (2014). No Difference in Small Bowel Microbiota between Patients with Irritable Bowel Syndrome and Healthy Controls. Sci. Rep..

[71] Andrews C.N., Sidani S., Marshall J.K. (2021). Clinical Management of the Microbiome in Irritable Bowel Syndrome. J. Can. Assoc. Gastroenterol..

[72] Rodiño-Janeiro B.K., Vicario M., Alonso-Cotoner C., Pascua-García R., Santos J. (2018). A Review of Microbiota and Irritable Bowel Syndrome: Future in Therapies. Adv. Ther..

[73] Asha M.Z., Khalil S.F.H. (2020). Efficacy and Safety of Probiotics, Prebiotics and Synbiotics in the Treatment of Irritable Bowel Syndrome a Systematic Review and Meta-Analysis. Sultan Qaboos Univ. Med. J..

[74] Accarie A., Vanuytsel T. (2020). Animal Models for Functional Gastrointestinal Disorders. Front. Psychiatry.

[75] Wang Y., Bi Z., Wang E., Sun B., Zheng Y., Zhong L., Yuan J. (2017). Rodent Model of Irritable Bowel Syndrome. Int. J. Gastroenterol. Disord. Ther..

[76] Fijan S. (2014). Microorganisms with Claimed Probiotic Properties: An Overview of Recent Literature. Int. J. Environ. Res. Public Health.

[77] McFarland L.V., Evans C.T., Goldstein J.C. (2018). Strain-Specificity and Disease-Specificity of Probiotic Efficacy: A Systematic Review and Meta-Analysis. Front. Med..

[78] Boyle R.J., Robins-Browne R.M., Tang M.L. (2006). Probiotic use in clinical practice: What are the risks?. Am. J. Clin. Nutr..

[79] Sen S., Mullan M.M., Parker T.J., Woolner J.T., Tarry S.A., Hunter J.O. (2002). Effect of *Lactobacillus plantarum* 299V on Colonic Fermentation and Symptoms of Irritable Bowel Syndrome. Dig. Dis. Sci..

[80] Ducrotté P., Sawant P., Jayanthi V. (2012). Clinical Trial: *Lactobacillus plantarum* 299V (DSM 9843) Improves Symptoms of Irritable Bowel Syndrome. World J. Gastroenterol..

[81] Pedersen S.M.M., Nielsen N.C., Andersen H.J., Olsson J., Simrén M., Öhman L., Svensson U., Malmendal A., Berthgam H.C. (2010). The Serum Metabolite Response to Diet Intervention with Probiotic Acidified Milk in Irritable Bowel Syndrome Patients Is Indistinguishable from That of Non-Probiotic Acidified Milk by 1H NMR-Based Metabonomic Analysis. Nutrients.

[82] Simrén M., Öhman L., Olsson J., Svensson U., Ohlson K., Posserud I., Strid H. (2010). Clinical Trial: The Effects of a Fermented Milk Containing Three Probiotic Bacteria in Patients with Irritable Bowel Syndrome—A Randomized, Double-Blind, Controlled Study. Aliment. Pharmacol. Ther..

[83] Søndergaard B., Olsson J., Ohlson K., Svensson U., Bytzer P., Ekesbo R. (2011). Effects of Probiotic Fermented Milk on Symptoms and Intestinal Flora in Patients with Irritable Bowel Syndrome: A Randomized, Placebo-Controlled Trial. Scand. J. Gastroenterol..

[84] Amirimani B., Nikfam S., Albaji M., Vahedi S., Nasseri-Moghaddam S., Sharafkhah M., Ansari R., Vahedi H. (2013). Probiotic vs. Placebo in Irritable Bowel Syndrome: A Randomized Controlled Trial. Middle East J. Dig. Dis..

[85] Roberts L.M., McCahon D., Holder R., Wilson S., Hobbs F.R. (2013). A Randomised Controlled Trial of a Probiotic “functional Food” in the Management of Irritable Bowel Syndrome. BMC Gastroenterol..

[86] Lorenzo-Zúñiga V., Llop E., Suárez C., Álvarez B., Abreu L., Espadaler J., Serra J. (2014). I.31, a New Combination of Probiotics, Improves Irritable Bowel Syndrome-Related Quality of Life. World J. Gastroenterol..

[87] Cremon C., Guglielmetti S., Gargari G., Taverniti V., Castellazzi A.M., Valsecchi C., Tagliacarne C., Fiore W., Bellini M., Bertani L. (2018). Effect of *Lactobacillus paracasei* CNCM I-1572 on Symptoms, Gut Microbiota, Short Chain Fatty Acids, and Immune Activation in Patients with Irritable Bowel Syndrome: A Pilot Randomized Clinical Trial. United Eur. Gastroenterol. J..

[88] Ligaarden S.C., Axelsson L., Naterstad K., Lydersen S., Farup P.G. (2010). A Candidate Probiotic with Unfavourable Effects in Subjects with Irritable Bowel Syndrome: A Randomised Controlled Trial. BMC Gastroenterol..

[89] Whorwell P.J., Altringer L., Morel J., Bond Y., Charbonneau D., O’Mahony L., Kiely B., Shanahan F., Quigley E.M.M. (2006). Efficacy of an Encapsulated Probiotic *Bifidobacterium infantis* 35,624 in Women with Irritable Bowel Syndrome. Am. J. Gastroenterol..

[90] Guglielmetti S., Mora D., Gschwender M., Popp K. (2011). Randomised Clinical Trial: *Bifidobacterium bifidum* MIMBb75 Significantly Alleviates Irritable Bowel Syndrome and Improves Quality of Life—A Double-Blind, Placebo-Controlled Study. Aliment. Pharmacol. Ther..

[91] Murakami K., Habukawa C., Nobuta Y., Moriguchi N., Takemura T. (2012). The Effect of *Lactobacillus brevis* KB290 against Irritable Bowel Syndrome: A Placebo-Controlled Double-Blind Crossover Trial. BioPsychoSoc. Med..

[92] Martoni C.J., Srivastava S., Leyer G.J. (2020). *Lactobacillus acidophilus* DDS-1 and *Bifidobacterium lactis* UABla-12 Improve Abdominal Pain Severity and Symptomology in Irritable Bowel Syndrome: Randomized Controlled Trial. Nutrients.

[93] Caviglia G.P., Tucci A., Pellicano R., Fagoonee S., Rosso C., Abate M.L., Olivero A., Armandi A., Vanni E., Saracco G.M. (2020). Clinical Response and Changes of Cytokines and Zonulin Levels in Patients with Diarrhoea-Predominant Irritable Bowel Syndrome Treated with *Bifidobacterium longum* ES1 for 8 or 12 Weeks: A Preliminary Report. J. Clin. Med..

[94] Zhou C., Fang X., Xu J., Gao J., Zhang L., Zhao J., Meng Y., Zhou W., Han X., Bai Y. (2020). *Bifidobacterium longum* Alleviates Irritable Bowel Syndrome-Related Visceral Hypersensitivity and Microbiota Dysbiosis via Paneth Cell Regulation. Gut Microbes.

[95] Lewis E.D., Antony J.M., Crowley D.C., Piano A., Bhardwaj R., Tompkins T.A., Evans M. (2020). Efficacy of *Lactobacillus paracasei* Ha-196 and *Bifidobacterium lngum* R0175 in Alleviating Symptoms of Irritable Bowel Syndrome (IBS): A Randomized, Placebo-Controlled Study. Nutrients.

[96] Kim J.Y., Park Y.J., Lee H.J., Park M.Y., Kwon O. (2018). Effect of *Lactobacillus gasseri* BNR17 on Irritable Bowel Syndrome: A Randomized, Double-Blind, Placebo-Controlled, Dose-Finding Trial. Food Sci. Biotechnol..

[97] Shin S.P., Choi Y.M., Kim W.H., Hong S.P., Park J.M., Kim J., Kwon O., Lee E.H., Hahm K.B. (2018). A Double Blind, Placebo-Controlled, Randomized Clinical Trial That Breast Milk Derived-*Lactobacillus gasseri* BNR17 Mitigated Diarrhea-Dominant Irritable Bowel Syndrome. J. Clin. Biochem. Nutr..

[98] Compare D., Rocco A., Coccoli P., Angrisani D., Sgamato C., Iovine B., Salvatore U., Nardone G. (2017). *Lactobacillus casei* DG and Its Postbiotic Reduce the Inflammatory Mucosal Response: An Ex-Vivo Organ Culture Model of Post-Infectious Irritable Bowel Syndrome. BMC Gastroenterol..

[99] Seong G., Lee S., Min Y.W., Jang Y.S., Kim H.S., Kim E.J., Park S.Y., Kim C.H., Chang D.K. (2021). Effect of Heat-Killed *Lactobacillus casei* DKGF7 on a Rat Model of Irritable Bowel Syndrome. Nutrients.

[100] Dapoigny M., Piche T., Ducrotte P., Lunaud B., Cardot J.M., Bernalier-Donadille A. (2012). Efficacy and Safety Profile of LCR35 Complete Freeze-Dried Culture in Irritable Bowel Syndrome: A Randomized, Double-Blind Study. World J. Gastroenterol..

[101] Urgesi R., Casale C., Pistelli R., Rapaccini G.L., de Vitis I. (2014). A Randomized Double-Blind Placebo-Controlled Clinical Trial on Efficacy and Safety of Association of Simethicone and *Bacillus coagulans* (Colinox^®^) in Patients with Irritable Bowel Syndrome. Eur. Rev. Med. Pharmacol. Sci..

[102] Majeed M., Nagabhushanam K., Natarajan S., Sivakumar A., Ali F., Pande A., Majeed S., Karri S.K. (2016). *Bacillus coagulans* MTCC 5856 Supplementation in the Management of Diarrhea Predominant Irritable Bowel Syndrome: A Double Blind Randomized Placebo Controlled Pilot Clinical Study. Nutr. J..

[103] Madempudi R.S., Ahire J.J., Neelamraju J., Tripathi A., Nanal S. (2019). Randomized Clinical Trial: The Effect of Probiotic *Bacillus coagulans* Unique IS2 vs. Placebo on the Symptoms Management of Irritable Bowel Syndrome in Adults. Sci. Rep..

[104] Gupta A.K., Maity C. (2021). Efficacy and Safety of *Bacillus coagulans* LBSC in Irritable Bowel Syndrome. Medicine.

[105] Sun Y.Y., Li M., Li Y.Y., Li L.X., Zhai W.Z., Wang P., Yang X.X., Gu X., Song L.J., Li Z. (2018). The Effect of *Clostridium butyricum* on Symptoms and Fecal Microbiota in Diarrhea-Dominant Irritable Bowel Syndrome: A Randomized, Double-Blind, Placebo-Controlled Trial. Sci. Rep..

[106] Zhao Q., Yang W.R., Wang X.H., Li G.Q., Xu L.Q., Cui X., Liu Y., Zuo X.L. (2019). *Clostridium butyricum* Alleviates Intestinal Low-Grade Inflamm TNBS-Induced Irritable Bowel Syndrome in Mice by Regulating Functional Status of Lamina Propria Dendritic Cells. World J. Gastroenterol..

[107] Kruis W., Chrubasik S., Boehm S., Stange C., Schulze J. (2012). A Double-Blind Placebo-Controlled Trial to Study Therapeutic Effects of Probiotic *Escherichia coli* Nissle 1917 in Subgroups of Patients with Irritable Bowel Syndrome. Int. J. Colorectal Dis..

[108] De Chambrun G.P., Neut C., Chau A., Cazaubiel M., Pelerin F., Justen P., Desreumaux P. (2015). A Randomized Clinical Trial of *Saccharomyces cerevisiae* versus Placebo in the Irritable Bowel Syndrome. Dig. Liver Dis..

[109] Spiller R., Pélerin F., Cayzeele Decherf A., Maudet C., Housez B., Cazaubiel M., Jüsten P. (2016). Randomized Double Blind Placebo-Controlled Trial of *Saccharomyces cerevisiae* CNCM I-3856 in Irritable Bowel Syndrome: Improvement in Abdominal Pain and Bloating in Those with Predominant Constipation. United Eur. Gastroenterol. J..

[110] Abbas Z., Yakoob J., Jafri W., Ahmad Z., Azam Z., Usman M.W., Shamim S., Islam M. (2014). Cytokine and Clinical Response to *Saccharomyces boulardii* Therapy in Diarrhea-Dominant Irritable Bowel Syndrome: A Randomized Trial. Eur. J. Gastroenterol. Hepatol..

[111] Hong K.B., Seo H., Lee J.S., Park Y. (2019). Effects of Probiotic Supplementation on Post-Infectious Irritable Bowel Syndrome in Rodent Model. BMC Complement. Altern. Med..

[112] Leventogiannis K., Gkolfakis P., Spithakis G., Tsatali A., Pistiki A., Sioulas A., Giamarellos-Bourboulis E.J., Triantafyllou K. (2019). Effect of a Preparation of Four Probiotics on Symptoms of Patients with Irritable Bowel Syndrome: Association with Intestinal Bacterial Overgrowth. Probiotics Antimicrob. Proteins.

[113] Barrett J.S., Canale K.E.K., Gearry R.B., Irving P.M., Gibson P.R. (2008). Probiotic Effects on Intestinal Fermentation Patterns in Patients with Irritable Bowel Syndrome. World J. Gastroenterol..

[114] Lee S.H., Joo N.S., Kim K.M., Kim K.N. (2018). The Therapeutic Effect of a Multistrain Probiotic on Diarrhea-Predominant Irritable Bowel Syndrome: A Pilot Study. Gastroenterol. Res. Pract..

[115] Sinn D.H., Song J.H., Kim H.J., Lee J.H., Son H.J., Chang D.K., Kim Y.H., Kim J.J., Rhee J.C., Rhee P.L. (2008). Therapeutic Effect of *Lactobacillus acidophilus*-SDC 2012, 2013 in Patients with Irritable Bowel Syndrome. Dig. Dis. Sci..

[116] Hong K.S., Kang H.W., Im J.P., Ji G.E., Kim S.G., Jung H.C. (2009). Effect of Probiotics on Symptoms in Korean Adults with Irritable Bowel Syndrome. Gut Liver.

[117] Michail S., Kenche H. (2011). Gut Microbiota Is Not Modified by Randomized, Double-Blind, Placebo-Controlled Trial of VSL#3 in Diarrhea-Predominant Irritable Bowel Syndrome. Probiotics Antimicrob. Proteins.

[118] Cui S., Hu Y. (2012). Multistrain Probiotic Preparation Significantly Reduces Symptoms of Irritable Bowel Syndrome in a Double-Blind Placebo-Controlled Study. Int. J. Clin. Exp. Med..

[119] Sisson G., Ayis S., Sherwood R.A., Bjarnason I. (2014). Randomised Clinical Trial: A Liquid Multi-Strain Probiotic vs. Placebo in the Irritable Bowel Syndrome—A 12 Week Double-Blind Study. Aliment. Pharmacol. Ther..

[120] Yoon H., Park Y.S., Lee D.H., Seo J.G., Shin C.M., Kim N. (2015). Effect of Administering a Multi-Species Probitic Mixture on the Change in Facial Microbiota and Symptoms of Irritable Bowel Syndrome: A Randomized, Double-Blind, Placebo-Controlled Trial. J. Clin. Biochem. Nutr..

[121] Mezzasalma V., Manfrini E., Ferri E., Sandionigi A., la Ferla B., Schiano I., Michelotti A., Nobile V., Labra M., di Gennaro P. (2016). A Randomized, Double-Blind, Placebo-Controlled Trial: The Efficacy of Multispecies Probiotic Supplementation in Alleviating Symptoms of Irritable Bowel Syndrome Associated with Constipation. BioMed Res. Int..

[122] Hod K., Dekel R., Aviv Cohen N., Sperber A., Ron Y., Boaz M., Berliner S., Maharshak N. (2018). The Effect of a Multispecies Probiotic on Microbiota Composition in a Clinical Trial of Patients with Diarrhea-Predominant Irritable Bowel Syndrome. Neurogastroenterol. Motil..

[123] Ishaque S.M., Khosruzzaman S.M., Ahmed D.S., Sah M.P. (2018). A Randomized Placebo-Controlled Clinical Trial of a Multi-Strain Probiotic Formulation (Bio-Kult^®^) in the Management of Diarrhea-Predominant Irritable Bowel Syndrome. BMC Gastroenterol..

[124] Oh J.H., Jang Y.S., Kang D., Chang D.K., Min Y.W. (2019). Efficacy and Safety of New Lactobacilli Probiotics for Unconstipated Irritable Bowel Syndrome: A Randomized, Double-Blind, Placebo-Controlled Trial. Nutrients.

[125] Zhang L., Liu Y.X., Wang Z., Wang X.Q., Zhang J.J., Jiang R.H., Wang X.Q., Zhu S.W., Wang K., Liu Z.J. (2019). Clinical Characteristic and Fecal Microbiota Responses to Probiotic or Antidepressant in Patients with Diarrhea-Predominant Irritable Bowel Syndrome with Depression Comorbidity: A Pilot Study. Chin. Med. J..

[126] Skrzydło-Radomańska B., Prozorow-Król B., Cichoż-Lach H., Majsiak E., Bierła J.B., Kanarek E., Sowińska A., Cukrowska B. (2021). The Effectiveness and Safety of Multi-Strain Probiotic Preparation in Patients with Diarrhea-Predominant Irritable Bowel Syndrome: A Randomized Controlled Study. Nutrients.

[127] Majeed M., Nagabhushanam K., Arumugam S., Majeed S., Ali F. (2018). *Bacillus coagulans* MTCC 5856 for the Management of Major Depression with Irritable Bowel Syndrome: A Randomised, Double-Blind, Placebo Controlled, Multi-Centre, Pilot Clinical Study. Food Nutr. Res..

[128] Olesen M., Gudmand-Høyer E. (2000). Efficacy, Safety, and Tolerability of Fructooligosaccharides in the Treatment of Irritable Bowel Syndrome. Am. J. Clin. Nutr..

[129] Azpiroz F., Dubray C., Bernalier-Donadille A., Cardot J.M., Accarino A., Serra J., Wagner A., Respondek F., Dapoigny M. (2017). Effects of ScFOS on the Composition of Fecal Microbiota and Anxiety in Patients with Irritable Bowel Syndrome: A Randomized, Double Blind, Placebo Controlled Study. Neurogastroenterol. Motil..

[130] Silk D.B.A., Davis A., Vulevic J., Tzortzis G., Gibson G.R. (2009). Clinical Trial: The Effects of a Trans-Galactooligosaccharide Prebiotic on Faecal Microbiota and Symptoms in Irritable Bowel Syndrome. Aliment. Pharmacol. Ther..

[131] Chen Q., Ren Y., Lu J., Bartlett M., Chen L., Zhang Y., Guo X., Liu C. (2017). A Novel Prebiotic Blend Product Prevents Irritable Bowel Syndrome in Mice by Improving Gut Microbiota and Modulating Immune Response. Nutrients.

[132] Niv E., Halak A., Tiommny E., Yanai H., Strul H., Naftali T., Vaisman N. (2016). Randomized Clinical Study: Partially Hydrolyzed Guar Gum (PHGG) versus Placebo in the Treatment of Patients with Irritable Bowel Syndrome. Nutr. Metab..

[133] Swanson K.S., Gibson G.R., Hutkins R., Reimer R.A., Reid G., Verbeke K., Scott K.P., Holscher H.D., Azad M.B., Delzenne N.M. (2020). The International Scientific Association for Probiotics and Prebiotics (ISAPP) Consensus Statement on the Definition and Scope of Synbiotics. Nat. Rev. Gastroenterol. Hepatol..

[134] Cappello C., Tremolaterra F., Pascariello A., Ciacci C., Iovino P. (2013). A Randomised Clinical Trial (RCT) of a Symbiotic Mixture in Patients with Irritable Bowel Syndrome (IBS): Effects on Symptoms, Colonic Transit and Quality of Life. Int. J. Colorectal Dis..

[135] Shavakhi A., Shavakhi S., Minakari M., Farzamnia S., Peykar M., Taghipour G., Tayebi A., Hashemi H. (2014). The Effects of Multi-Strain Probiotic Compound on Symptoms and Quality-of-Life in Patients with Irritable Bowel Syndrome: A Randomized Placebo-Controlled Trial. Adv. Biomed. Res..

[136] Bogovič Matijašic B., Obermajer T., Lipoglavšek L., Sernel T., Locatelli I., Kos M., Šmid A., Rogelj I. (2016). Effects of Synbiotic Fermented Milk Containing *Lactobacillus acidophilus* La-5 and *Bifidobacterium animalis* ssp. lactis BB-12 on the Fecal Microbiota of Adults with Irritable Bowel Syndrome: A Randomized Double-Blind, Placebo-Controlled Trial. J. Dairy Sci..

[137] Bucci C., Tremolaterra F., Gallotta S., Fortunato A., Cappello C., Ciacci C., Iovino P. (2014). A Pilot Study on the Effect of a Symbiotic Mixture in Irritable Bowel Syndrome: An Open-Label, Partially Controlled, 6-Month Extension of a Previously Published Trial. Tech. Coloproctol..

[138] Asgarshirazi M., Shariat M., Dalili H. (2015). Comparison of the Effects of Ph-Dependent Peppermint Oil and Synbiotic Lactol (*Bacillus coagulans* + Fructooligosaccharides) on Childhood Functional Abdominal Pain: A Randomized Placebo-Controlled Study. Iran. Red Crescent Med. J..

[139] Moser A.M., Spindelboeck W., Halwachs B., Strohmaier H., Kump P., Gorkiewicz G., Högenauer C. (2019). Effects of an Oral Synbiotic on the Gastrointestinal Immune System and Microbiota in Patients with Diarrhea-Predominant Irritable Bowel Syndrome. Eur. J. Nutr..

[140] Lee S.H., Cho D.Y., Lee S.H., Han K.S., Yang S.W., Kim J.H., Lee S.H., Kim S.M., Kim K.N. (2019). A Randomized Clinical Trial of Synbiotics in Irritable Bowel Syndrome: Dose-Dependent Effects on Gastrointestinal Symptoms and Fatigue. Korean J. Fam. Med..

[141] Min Y.W., Park S.U., Jang Y.S., Kim Y.H., Rhee P.L., Ko S.H., Joo N., Kim S.I., Kim C.H., Chang D.K. (2012). Effect of Composite Yogurt Enriched with Acacia Fiber and *Bifidobacterium lactis*. World J. Gastroenterol..

[142] Bahrudin M.F., Abdul Rani R., Tamil A.M., Mokhtar N.M., Raja Ali R.A. (2020). Effectiveness of Sterilized Symbiotic Drink Containing *Lactobacillus helveticus* Comparable to Probiotic Alone in Patients with Constipation-Predominant Irritable Bowel Syndrome. Dig. Dis. Sci..

[143] Skrzydło-Radomańska B., Prozorow-Król B., Cichoż-Lach H., Majsiak E., Bierła J.B., Kosikowski W., Szczerbiński M., Gantzel J., Cukrowska B. (2020). The Effectiveness of Synbiotic Preparation Containing *Lactobacillus* and *Bifidobacterium* Probiotic Strains and Short Chain Fructooligosaccharides in Patients with Diarrhea Predominant Irritable Bowel Syndrome—A Randomized Double-Blind, Placebo-Controlled Study. Nutrients.

[144] Seong G., Lee S., Min Y.W., Jang Y.S., Park S.Y., Kim C.H., Lee C., Hong S.N., Chang D.K. (2020). Effect of a Synbiotic Containing *Lactobacillus paracasei* and *Opuntia Humifusa* on a Murine Model of Irritable Bowel Syndrome. Nutrients.

